# Exploring the Anticancer Potential of NO-Donor Oxadiazole Assemblies Against Malignant Pleural Mesothelioma

**DOI:** 10.3390/pharmaceutics17020230

**Published:** 2025-02-10

**Authors:** Irina A. Stebletsova, Alexander A. Larin, Egor M. Matnurov, Ivan V. Ananyev, Maria V. Babak, Leonid L. Fershtat

**Affiliations:** 1 N.D. Zelinsky Institute of Organic Chemistry, Russian Academy of Sciences, 47 Leninsky Prosp., 119991 Moscow, Russia; irinastebl@icloud.com (I.A.S.); roby3@mail.ru (A.A.L.); 2 Higher Chemical College of the Russian Academy of Science, D.I. Mendeleev University of Chemical Technology of Russia, 9 Miusskaya Square, 125047 Moscow, Russia; 3 Drug Discovery Lab, Department of Chemistry, City University of Hong Kong, 83 Tat Chee Avenue, Hong Kong SAR 999077, China; ematnurov2-c@my.cityu.edu.hk; 4 N.S. Kurnakov Institute of General and Inorganic Chemistry, Russian Academy of Sciences, 31 Leninsky Prosp., 119991 Moscow, Russia; ananyev@ineos.ac.ru

**Keywords:** nitrogen heterocycles, furoxan, oxadiazole, malignant pleural mesothelioma, NO donors, antiproliferative activity

## Abstract

**Background:** Nitric oxide (NO) has been linked to the pathogenesis of asbestos-related pleural diseases, including an extremely aggressive cancer called malignant pleural mesothelioma (MPM). Given that MPM cells are characterized by a higher expression of NO synthases and elevated NO production relative to normal cells, the use of NO-donor compounds could potentially saturate the cancerous cells with NO, triggering their death. **Methods**: We developed a novel class of NO prodrugs by merging two NO-releasing components, 1,2,5-oxadiazole 2-oxides (furoxans) and 1,2,4-oxadiazoles, and studied their NO-releasing characteristics in a time-dependent manner using the Griess assay. The cytotoxicity against two human MPM cell lines and non-cancerous lung fibroblasts was evaluated using a colorimetric MTT assay. **Results**: All compounds exhibited excellent NO-donating properties, surpassing the capacity of two reference NO donor compounds, 3-carbamoyl-4-(hydroxymethyl)furoxan (CAS-1609) and 4-ethoxy-3-phenylsulphonylfuroxan (CHF-2363), by at least 1.5–3 times. All oxadiazole hybrids demonstrated high cytotoxicity against MPM cell lines in a low micromolar range, comparable or higher than the cytotoxicity of the standard-of-care drug cisplatin. **Conclusions**: Notably, the novel compounds displayed a markedly greater selectivity towards cancerous cells than cisplatin when compared with non-cancerous lung fibroblasts, aligning with the intended design.

## 1. Introduction

Malignant pleural mesothelioma (MPM) is a rare and aggressive cancer that primarily affects the lining of the lungs, called pleura [1]. This malignancy is notorious for its poor prognosis and rapid progression, often leading to devastating outcomes for patients [2]. The primary cause of MPM is the inhalation of asbestos fibers, which can trigger chronic inflammation and genetic mutations [3]. Despite advances in treatment, including surgery, chemotherapy, and emerging immunotherapies, the survival rates remain less than 5% [4]. Hence, novel therapeutic strategies are urgently required to address MPM effectively, and one of the emerging strategies involves overloading malignant cells with nitric oxide (NO) [5].

Nitric oxide (NO) has a multifaceted impact on the pleural cavity, as it was shown to induce both cytotoxic and proliferative signals, depending on the NO concentration and duration of exposure [6]. For example, the cytoprotective role of NO in the pleural cavity was related to the mediation of essential physiological functions and immune responses, while an excess of NO was linked to the induction of genotoxic and mutagenic effects [6], potentially resulting in tumor growth and progression [7].

In particular, the exposure of pleural cells to asbestos has been associated with an increase in cellular NO levels through the activation of NO synthases. Consequently, MPM cells often exhibit elevated expression levels of NO synthases, including both inducible and endothelial forms [6]. Notably, epithelial MPM subtypes generally exhibit higher expression compared to sarcomatoid subtypes [8]. The upregulation of NO synthases serves to enhance the proliferative and survival responses of MPM cells [6,9]. In contrast, excessive cellular concentrations of NO can directly trigger cancer cell death [10]. Consequently, NO donors have the potential to act as anticancer agents by overwhelming MPM cells with already elevated NO levels [11].

1,2,5-Oxadiazole 2-oxides, also known as furoxans, release NO upon activation by intracellular thiols, thereby functioning as NO prodrugs [12]. They have attracted substantial interest as NO donors due to their beneficial pharmacological profile, including a slow onset, prolonged action, and high efficacy against various tumor types both in vitro and in vivo [12,13,14,15]. The hybridization of furoxans with other pharmacophoric scaffolds resulted in improved efficacy and dual mechanisms of action, thereby offering promise in addressing the multidrug resistance issue [14,16].

**Figure 1:**
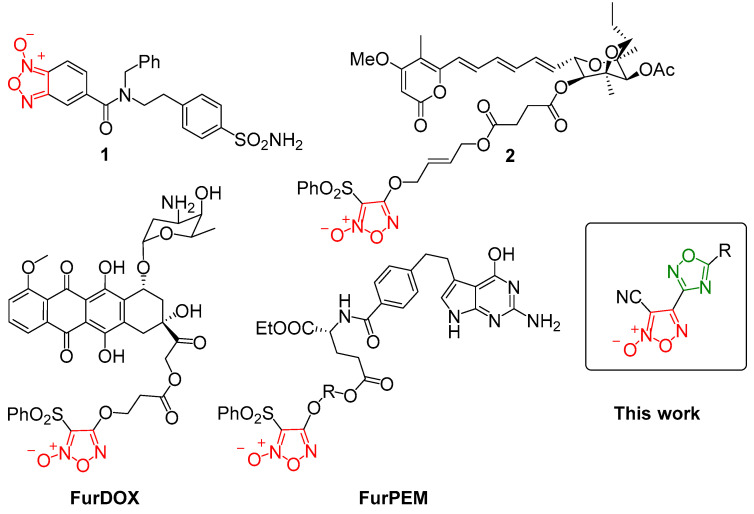
Furoxan-based derivatives for anticancer treatment. Furoxan fragments are depicted in red color; 1,2,4-oxadiazole fragment is depicted in green color.

For example, benzofuroxan conjugate with a carbonic anhydrase inhibitor SLC-0111 exhibited high antiproliferative activity against renal carcinoma A-498 cells [17], while furoxan–aurovertin hybrid **2** displayed potent in vitro and in vivo effects against triple-negative breast cancer, acting as a dual ferroptosis and apoptosis inducer (Figure 1) [18]. In another study, 3-phenylsulfonylfuroxan–doxorubicin hybrid (**FurDOX**, Figure 1) effectively overcame drug resistance in a doxorubicin-resistant human colon cell line HT29-dx by diminishing the activity of ATP-binding cassette transporters that typically eject the drug [16].

Surprisingly, despite the well-documented NO-releasing properties of furoxans, studies investigating the efficacy of furoxan derivatives against MPM are notably scarce. To the best of our knowledge, **FurDOX** is the only furoxan derivative that has been directly tested against malignant mesothelioma MM98 cells and non-malignant mesothelioma Met5A cells [16]. This compound demonstrated enhanced intracellular accumulation in both types of cells in comparison with unmodified doxorubicin. However, as doxorubicin is not commonly used for treating MPM, the authors proceeded with a thorough exploration of **FurDOX** in the context of colon cancer [16]. In another study, Min et al. prepared a series of compounds based on the conjugation of 3-phenylsulfonylfuroxan derivatives with pemetrexed [19], a standard-of-care first-line systemic treatment for mesothelioma (**FurPEM**, Figure 1) [20]. The **FurPEM** derivatives exhibited some antiproliferative effects in four cancer cell lines, including BGC, HL60, SMMC, and A549. However, these derivatives have not been evaluated against any MPM cell lines [20]. This highlights a significant research gap in understanding the potential of furoxan derivatives in the treatment of MPM. To bridge this gap, we developed a novel class of furoxan hybrids, integrating 1,2,4-oxadiazole as an additional NO-donating component (Figure 1), and extensively studied their cytotoxicity, NO-releasing characteristics, and structure–activity correlations, specifically focusing on MPM.

## 2. Experimental Section

### 2.1. General Information

All reactions were carried out in well-cleaned oven-dried glassware with magnetic stirring. ^1^H and ^13^C NMR spectra were recorded on a Bruker AM-300 (300.13 and 75.47 MHz, respectively) spectrometer (Bruker Corporation, Billerica, MA, USA) and referenced to a residual solvent peak. The chemical shifts are reported in ppm (δ). Mass spectra were measured using an INCOS-50 instrument (Finnigan MAT Co., San Jose, CA, USA). The IR spectra were recorded on a Simex FT-801 IR-Fourier spectrometer (Simex, Novosibirsk, Russia) in the 4000–550 cm^−1^ region (spectral resolution 4 cm^−1^), using the universal optical attenuated total reflection (ATR) accessory with a ZnSe crystal plate. ZaIR 3.5 software (Simex, Novosibirsk, Russia) was used to carry out baseline correction and normalization of the FEAR spectra. A background (air) measurement was taken for every sample processed. The peaks corresponding to CO_2_ vibrations were removed using the “straight line generation” option in the ZaIR 3.5 software (Simex, Novosibirsk, Russia). Raw spectra were preprocessed using a simple two-point linear subtraction baseline correction method. Spectra were then vector normalized. Spectrum smoothing was not performed. High-resolution mass spectra were recorded on a Bruker microTOF spectrometer (Bruker Corporation, Billerica, MA, USA) with electrospray ionization (ESI). All measurements were performed in positive (+MS) ion mode (interface capillary voltage: 4500 V) with a scan range of m/z: 50–3000. External calibration of the mass spectrometer was performed with Electrospray Calibrant Solution (Fluka, Buchs, Switzerland). A direct syringe injection was used for all analyzed solutions in MeCN or CH_3_OH (flow rate: 3 μL min^−1^). Nitrogen was used as nebulizer gas (0.4 bar) and dry gas (4.0 L∙min^−1^); the interface temperature was set to 180 °C. All spectra were processed by using a Bruker Data Analysis 4.0 software package (Billerica, MA, USA). Elemental analyses were performed by using the CHN Analyzer Perkin-Elmer 2400 (Perkin Elmer, Norwalk, CT, USA). Analytical thin-layer chromatography (TLC) was carried out on Merck 25 TLC silica gel 60 F254 aluminum sheets (E. Merck, Darmstadt, Germany). The visualization of the TLC plates was accomplished with a UV light. All solvents were purified and dried using standard methods prior to use. All standard reagents were purchased from (Steinheim, Germany) or Acros Organics (NJ, USA) and were used without further purification. Some products were purified via column chromatography. Chromatography was performed on silica gel 60 Å (0.060–0.200 mm, Acros Organics, NJ, USA).

### 2.2. Synthesis of 3-Cyano-(1,2,4-Oxadiazol-3-yl)Furoxans (General Procedure)

1,1-Carbonyldiimidazole (CDI) (1.05 mmol, 170 mg) was added to a solution of the corresponding carboxylic acid (1.05 mmol) in acetonitrile (10 mL). The reaction mixture was stirred for 1 h at room temperature. Furoxanylamidoxime **1** (1 mmol, 169 mg) was added to the formed acylimidazole and the resulting mixture was additionally stirred for 3 h until the complete formation of acylated intermediates was achieved. The reaction progress was monitored by TLC. Then, 1,4-diazabicyclo[2.2.2]octane (DABCO) (2 mmol, 224 mg) was added, and the reaction mixture was heated to 50 °C and stirred for 12–52 h until complete formation of the cyclization products (**2a**–**k**). Then, the reaction mixture was poured into water (15 mL) and the precipitate formed was filtered off and dried in air.

**3-Cyano-4-(5-phenyl-1,2,4-oxadiazolyl)furoxan, 2a:** yield 181 mg (71%), white powder; R*_f_* (15 CHCl_3_:1 EtOAc) = 0.82. ^1^H NMR (300 MHz, DMSO-[d_6_]) *δ*, ppm: 8.24 (dd, *J* = 8.3, 1.3 Hz, 2H), 7.85–7.80 (m, 1H), 7.76–7.70 (m, 2H); ^13^C NMR (75 MHz, DMSO-[d_6_]) *δ*, ppm: 158.9, 151.3, 146.2, 134.9, 130.3, 128.9, 122.7, 106.5, 98.3. IR (KBr), *ν*: 2360, 2341, 2258, 1621, 1605, 1558, 1451, 1438, 1362, 1258, 1033, 958, 932, 839, 747, 709, 684 cm^−1^. HRMS (ESI) calcd. for C_11_H_6_N_5_O_3_^+^: 256.0465. Found: 256.0468 [M+H]^+^.

**3-Cyano-4-(5-(4-nitrophenyl)-1,2,4-oxadiazolyl)furoxan, 2b:** yield 169 mg (56%), cream powder; R*_f_* (15 CHCl_3_:1 EtOAc) = 0.9. ^1^H NMR (300 MHz, DMSO-[d_6_]) *δ*, ppm: 8.54–8.48 (m, 4H); ^13^C NMR (75 MHz, DMSO-[d_6_]) *δ*, ppm: 176.2, 159.1, 151.0, 145.9, 130.5, 128.2, 125.3, 106.4, 98.3. IR (KBr), *ν*: 2360, 2341, 2254, 1649, 1564, 1529, 1411, 1359, 1343, 1255, 1034, 958, 879, 838, 746, 711 cm^−1^. HRMS (ESI) calcd. for C_11_H_5_N_6_O_5_^+^: 301.0316. Found: 301.0315 [M+H]^+^.

**3-Cyano-4-(5-(3-nitrophenyl)-1,2,4-oxadiazolyl)furoxan, 2c:** yield 144 mg (48%), cream powder; R*_f_* (15 CHCl_3_:1 EtOAc) = 0.9. ^1^H NMR (300 MHz, DMSO-[d_6_]) *δ*, ppm: 8.88 (s, 1H), 8.65 (t, *J* = 8.0 Hz, 2H), 8.03 (t, *J* = 8.1 Hz, 1H); ^13^C NMR (75 MHz, Acetone-[d_6_]) *δ*, ppm: 176.0, 159.1, 148.9, 145.5, 134.1, 131.5, 128.3, 124.5, 123.1, 105.4, 97.0. IR (KBr), *ν*: 2251, 1654, 1623, 1534, 1447, 1351, 1281, 823, 738, 711 cm^−1^. HRMS (ESI) calcd. for C_11_H_5_N_6_O_5_^+^: 301.0316. Found: 301.0313 [M+H]^+^.

**3-Cyano-4-(5-(2-nitrophenyl)-1,2,4-oxadiazolyl)furoxan, 2d:** yield 123 mg (41%), cream powder; R*_f_* (15 CHCl_3_:1 EtOAc) = 0.9. ^1^H NMR (300 MHz, DMSO-[d_6_]) *δ*, ppm: 8.36–8.33 (m, 1H), 8.25–8.22 (m, 1H), 8.10–8.04 (m, 2H); ^13^C NMR (75 MHz, Acetone-[d_6_]) *δ*, ppm: 175.2, 158.9, 150.3, 146.2, 134.7, 134.0, 132.1, 131.0, 125.3, 105.3, 98.7. IR (KBr), *ν*: 2245, 1621, 1565, 1537, 1504, 1349, 937, 834, 747 cm^−1^. HRMS (ESI) calcd. for C_11_H_8_N_7_O_5_^+^: 318.0581; for C_11_H_4_N_6_O_5_Na^+^: 323.0135. Found: 318.0573 [M+NH_4_]^+^, 323.0128 [M+Na]^+^.

**3-Cyano-4-(5-(pyridin-4-yl)-1,2,4-oxadiazolyl)furoxan, 2e:** yield 95 mg (37%), white solid; R*_f_* (10 CHCl_3_:1 EtOAc) = 0.63. ^1^H NMR (300 MHz, DMSO-[d_6_]) *δ*, ppm: 8.97 (dd, *J* = 6.1, 1.7 Hz, 2H), 8.16 (dd, *J* = 6.1, 1.7 Hz, 2H); ^13^C NMR (75 MHz, DMSO-[d_6_]) *δ*, ppm: 176.2, 159.1, 151.9, 146.0, 129.9, 122.0, 106.4, 98.3. IR (KBr), *ν*: 2255, 1644, 1572, 1547, 1439, 1409, 1360, 1254, 1111, 1035, 958, 923, 848, 757, 709, 696, 669 cm^−1^. HRMS (ESI) calcd. for C_10_H_5_N_6_O_3_^+^: 257.0418. Found: 257.0415 [M+H]^+^.

**3-Cyano-4-(5-(methoxymethyl)-1,2,4-oxadiazolyl)furoxan, 2f:** yield 181 mg (81%), pale orange solid; R*_f_* (1 CHCl_3_:1 EtOAc) = 0.47. ^1^H NMR (300 MHz, DMSO-[d_6_]) *δ*, ppm: 4.97 (s, 2H), 3.47 (s, 3H); ^13^C NMR (75 MHz, DMSO-[d_6_]) *δ*, ppm: 179.4, 158.2, 145.0, 106.4, 98.3, 64.9, 59.5. IR (KBr), *ν*: 2936, 2832, 2360, 2341, 2257, 1712, 1619, 1487, 1446, 1356, 1292, 1253, 1194, 1112, 1071, 1021, 957, 925, 829, 686 cm^−1^. HRMS (ESI) calcd. for C_7_H_6_N_5_O_4_·H_2_O^+^: 242.0520; for C_7_H_5_N_5_O_4_Na·H_2_O^+^: 264.0339. Found: 242.0524 [M+H_2_O+H]^+^, 264.0339 [M+H_2_O+Na]^+^.

**3-Cyano-4-(5-(*p*-tolyl)-1,2,4-oxadiazolyl)furoxan, 2g:** yield 137 mg (51%), cream powder; R*_f_* (15 CHCl_3_:1 EtOAc) = 0.64. ^1^H NMR (300 MHz, DMSO-[d_6_]) *δ*, ppm: 8.13 (d, *J* = 8.3 Hz, 2H), 7.54 (d, *J* = 7.9 Hz, 2H), 2.46 (s, 3H); ^13^C NMR (75 MHz, DMSO-[d_6_]) *δ*, ppm: 177.6, 158.8, 146.2, 145.6, 130.9, 128.8, 120.0, 106.5, 98.3, 21.8. IR (KBr), *ν*: 2987, 2244, 1632, 1560, 1440, 1359, 1251, 1104, 1047, 955, 827, 756, 725 cm^−1^. Calcd. for C_12_H_7_N_5_O_3_ (%): C, 53.54; H, 2.62; N, 26.01. Found (%): C, 53.65; H, 2.51; N, 25.87.

**3-Cyano-4-(5-(4-methoxyphenyl)-1,2,4-oxadiazolyl)furoxan, 2h:** yield 165 mg (58%), pink powder; R*_f_* (15 CHCl_3_:1 EtOAc) = 0.5. ^1^H NMR (300 MHz, DMSO-[d_6_]) *δ*, ppm: 8.18 (d, *J* = 8.5 Hz, 2H), 7.24 (d, *J* = 7.4 Hz, 2H), 3.91 (s, 3H); ^13^C NMR (75 MHz, DMSO-[d_6_]) *δ*, ppm: 177.4, 164.4, 158.7, 146.3, 131.0, 115.8, 114.9, 106.5, 98.3, 56.3. IR (KBr), *ν*: 2971, 2216, 1600, 1507, 1427, 1259, 1177, 1096, 1019, 810, 759 cm^−1^. HRMS (ESI) calcd. for C_12_H_8_N_5_O_4_^+^: 286.0571. Found: 286.0570 [M+H]^+^.

**3-Cyano-4-(5-(3,4-dimethoxyphenyl)-1,2,4-oxadiazolyl)furoxan, 2i:** yield 202 mg (64%), pink powder; R*_f_* (15 CHCl_3_:1 EtOAc) = 0.83. ^1^H NMR (300 MHz, DMSO-[d_6_]) *δ*, ppm: 7.85 (d, *J* = 8.7 Hz, 1H), 7.65 (s, 1H), 7.27 (d, *J* = 8.5 Hz, 1H), 3.90 (s, 6H); ^13^C NMR (75 MHz, DMSO-[d_6_]) *δ*, ppm: 177.4, 158.8, 154.3, 149.7, 146.2, 123.2, 114.7, 112.7, 110.8, 106.5, 98.2, 56.4, 56.3. IR (KBr), *ν*: 2947, 2248, 1632, 1605, 1560, 1509, 1485, 1433, 1362, 1281, 1257, 1230, 1147, 1018, 958, 869, 827, 762 cm^−1^. HRMS (ESI) calcd. for C_13_H_10_N_5_O_5_^+^: 316.0676. Found: 316.0677 [M+H]^+^.

**4-(5-(2-Chlorophenyl)-1,2,4-oxadiazolyl)-3-cyanofuroxan, 2j:** yield 168 mg (58%), cream powder; R*_f_* (1 CHCl_3_:3 EtOAc) = 0.92. ^1^H NMR (300 MHz, DMSO-[d_6_]) *δ*, ppm: 8.26 (d, *J* = 7.3 Hz, 1H), 7.82 (d, *J* = 6.6 Hz, 2H), 7.71–7.66 (m, 1H); ^13^C NMR (75 MHz, DMSO-[d_6_]) *δ*, ppm: 176.0, 158.6, 146.1, 135.7, 133.2, 133.0, 132.2, 128.7, 121.9, 106.4, 98.4. IR (KBr), *ν*: 2242, 1608, 1556, 1498, 1432, 1329, 1274, 1182, 1026, 975, 946, 831, 807, 757, 729 cm^−1^. Calcd. for C_11_H_4_ClN_5_O_3_ (%): C, 45.62; H, 1.39; N, 24.18. Found (%): C, 45.43; H, 1.51; N, 23.99.

**3-Cyano-4-(5-(2-furyl)-1,2,4-oxadiazolyl)furoxan, 2k:** yield 186 mg (76%), dark pink powder; R*_f_* (15 CHCl_3_:1 EtOAc) = 0.8. ^1^H NMR (300 MHz, Acetone-[d_6_]) *δ*, ppm: 8.12 (dd, *J* = 1.8, 0.8 Hz, 1H), 7.73 (dd, *J* = 3.7, 0.8 Hz, 1H), 6.92 (dd, *J* = 3.7, 1.8 Hz, 1H); ^13^C NMR (75 MHz, Acetone-[d_6_]) *δ*, ppm: 169.2, 158.6, 149.0, 145.6, 138.9, 119.1, 113.3, 106.4, 93.6. IR (KBr), *ν*: 3133, 2247, 1632, 1523, 1450, 1360, 1103, 1019, 958, 789 cm^−1^. HRMS (ESI) calcd. for C_9_H_3_N_5_O_4_Na^+^: 268.0077. Found: 268.0079 [M+Na]^+^.

### 2.3. Synthesis of 3-Cyano-4-(1,2,4-Oxadiazol-3-yl)Furoxan

Furoxanylamidoxime **1** (1.5 mmol, 254 mg) and *p*-toluene sulfonic acid monohydrate (PTSA) (0.15 mmol, 29 mg) were added to a vigorously stirred solution of trimethyl orthoformate (10 mmol, 1.1 mL) in acetonitrile (5 mL). The reaction mixture was stirred for 12 h at room temperature until the consumption of substrate **1** (TLC monitoring, eluent CHCl_3_/EtOAc, 1:1). The reaction mixture was poured into a glass containing 1/3 CH_2_Cl_2_ and 2/3 ice, and it was extracted with CH_2_Cl_2_. Combined organic layers were washed with H_2_O and dried over MgSO_4_. The solvent was driven off at reduced pressure on a rotary evaporator. The target product was additionally purified by column chromatography (eluent CHCl_3_:EtOAc, 1:1).

**3-Cyano-4-(1,2,4-oxadiazolyl)furoxan, 2l:** yield 177 mg (66%), colorless oil; R*_f_* (15 CHCl_3_:1 EtOAc) = 0.54. ^1^H NMR (300 MHz, DMSO-[d_6_]) *δ*, ppm: 10.14 (s, 1H); ^13^C NMR (75 MHz, DMSO-[d_6_]) *δ*, ppm: 169.8, 157.6, 146.0, 106.4, 98.3. IR (KBr), *ν*: 3136, 2250, 1620, 1530, 1472, 1349, 1283, 1094, 1045, 1031, 907, 829, 756, 684 cm^−1^. HRMS (ESI) calcd. for C_5_HN_5_O_3_Ag: 285.9125 (^107^Ag), 287.9122 (^109^Ag). Found: 285.9123 (^107^Ag), 287.9120 (^109^Ag) [M+Ag]^+^.

### 2.4. NO Release Assay

The test molecule (0.1 mmol) was dissolved in DMSO (50 mL). Then, 20 μL aliquot of the resulting solution was diluted with phosphate buffer solution (180 μL, pH 7.4, containing 2 μmol *L*-cysteine). The final concentration of the tested compound was 2 × 10^−4^ M. The mixture was incubated at 37 °C for 1 h. Then, a 50 μL aliquot of Griess reagent (prepared by mixing sulfanilamide (4 g), *N*-naphthylethylenediamine dihydrochloride (0.2 g), and 85% H_3_PO_4_ (10 mL) in distilled and deionized water (final volume 100 mL)) was added and incubated for 10 min at 37 °C. UV absorbance at 540 nm was measured using a Multiskan GO Microplate Photometer (Thermo Scientific, New York, NY, USA) and calibrated using a standard curve prepared from standard solutions of NaNO_2_ to give the nitrite concentration. All measurements were made in triplicate.

### 2.5. X-Ray Diffraction Study

The molecular structure of **2a** was ascertained using a Bruker Quest diffractometer (MoKa-radiation, graphite monochromator, ω-scans) equipped with a Photon-II area-detector at the facilities of the JRC PMR IGIC RAS. The intensity data were integrated by the SAINT program (version 8.40B) [21] and were corrected for absorption and decay using SADABS [22]. Both structures were solved by dual-space methods using SHELXT 2014/6 [23] and refined on F^2^ using SHELXL-2018 [24]. The positions of hydrogen atoms were found from difference Fourier synthesis and then refined by invoking isotropic approximation. All non-hydrogen atoms were refined with individual anisotropic displacement parameters. The main crystallography data and refinement parameters are given in the Table S2.1. The CCDC number 2388378 contains all the additional information on the crystal structure and the details of refinement. The full optimization of the structures of the isolated molecule of **2a** were performed using the Gaussian 09 program (rev. D01) [25] at the PBE0 [26,27] /def2TZVP level with the Grimme’s D3 dispersion corrections and Becke–Jonson damping [28]. Tight convergence criteria were used for the optimization procedures. The equilibrium structures of compound correspond to minimums on the potential energy surface according to the calculations of the Hessian of electronic energy (ultrafine grids, no imaginary modes were found). The root mean squared difference between the optimized and crystal conformation was calculated, with the positions of hydrogen atoms being optimized at the same level for crystal conformation.

### 2.6. Cell Lines and Culture Conditions

AB1 (mouse malignant mesothelioma cell line) and JU77 (human mesothelioma cell line) were obtained from Cell Bank Australia; MRC-5 (human embryonic lung fibroblast cell line) was obtained from ATCC. AB1 and JU77 were cultured in RPMI containing 10% FBS and 1% penicillin–streptomycin (10,000 U mL^−1^); MRC-5 was cultured in MEM containing 10% FBS, 1% penicillin–streptomycin (10,000 U mL^−1^), and 1% L-glutamine (200 mM). Cells were grown in tissue culture flasks (75 cm^2^ and 25 cm^2^, SPL Life Sciences, Pocheon, South Korea). All cell lines were grown at 37 °C in a humidified atmosphere containing 95% air and 5% CO_2_. All drug stock solutions were prepared in DMSO, and the final concentration in the medium did not exceed 1%, at which cell viability was not inhibited.

### 2.7. Evaluation of Anticancer Activity

The cytotoxicity of compounds was determined using the MTT colorimetric test. Cells were harvested from culture flasks by trypsinization and seeded into Cellstar 96-well microculture plates at a seeding density of 6000 cells per well (6 × 10^4^ cells per mL). After the cells were allowed to resume exponential growth for 24 h, they were exposed to drugs at different concentrations in media for 72 h. The tested compounds were diluted in the complete medium at the desired concentrations added to each well (100 μL) and serially diluted to other wells. After 72 h of exposure, the medium was replaced with MTT (5 mg mL^−1^, 100 μL per well) and incubated for an additional 50 min. Subsequently, the medium was aspirated, and the purple formazan crystals formed in viable cells were dissolved in DMSO (100 μL per well). Optical densities were measured at 570 nm using a Multiskan SkyHigh Microplate Spectrophotometer (Life Technologies Holdings Pte., Singapore) The quantity of viable cells was expressed in terms of treated/control (T/C) values by comparison to untreated control cells, and 50% inhibitory concentrations (IC_50_) were calculated by interpolation from concentration–effect curves. The evaluation was based on means from at least three independent experiments, each comprising three replicates per concentration level.

### 2.8. Evaluation of NO Release In Vitro

The NO release of compounds in vitro was determined using Griess reagent from a Nitrite/Nitrate Assay colorimetric kit (Sigma-Aldrich, Steinheim, Germany). Cells were harvested from culture flasks by trypsinization and seeded into Cellstar 96-well microculture plates at a seeding density of 10,000 cells per well (1 × 10^5^ cells per mL). After the cells were allowed to resume exponential growth for 24 h, they were exposed to 100 μL of drugs at a concentration of 25 μM in colorless RPMI media (Gibco, UK) for 24 h. After exposure for 24 h, 10 μL of Griess reagent A was added to each well and the plate was mixed using a horizontal shaker at room temperature for 5 min. Then, 10 μL of Griess reagent B was added to each well and the plate was mixed using a horizontal shaker at room temperature for 15 min. NO release presented as a fold change normalized to an untreated control (no drug), while NaNO_2_ standard solution from the kit at concentrations 0, 2, 4, and 8 nM (100 μL per well) was prepared as the positive control for the standard curve for each experiment. The evaluation was based on the mean from at least three independent experiments, each comprising three replicates per concentration level.

## 3. Results and Discussion

### 3.1. Synthesis and Characterization

Previously, we developed a synthetic approach towards mono- and bis(1,2,4-oxadiazolyl)furoxans based on the tandem heterocyclization of furoxanylamidoximes with various carboxylic acid chlorides [29]. However, this approach required the preliminary isolation of moderately stable carboxylic acid chlorides. Therefore, in this study, we aimed to devise a more straightforward synthetic method based on the reaction of furoxanylamidoximes with carboxylic acids. Initially, we optimized the reaction conditions using furoxanylamidoxime **1** and benzoic acid as the selected model substrates. Different activation reagents, bases, reaction times, and temperatures were systematically screened to determine the overall efficacy of the synthetic protocol (Table 1). The use of the toxic reagent SOCl_2_ as an activating reagent and Cs_2_CO_3_ or triethylamine (TEA) as the base resulted in moderate yields of **2a** (entries 1–6). In contrast, the use of 1,1′-carbonyldiimidazole (CDI) as an activating reagent was more effective, in particular when using 1,4-diazabicyclo[2.2.2]octane (DABCO) as the base in comparison to 1,8-diazabicyclo[5.4.0]undec-7-ene (DBU) or TEA (entries 7–10). Therefore, the optimal conditions for the synthesis of **2a** included the utilization of CDI as an activating reagent and DABCO as a base for 3 h at 50 °C (entry 10).

**Scheme 1:**
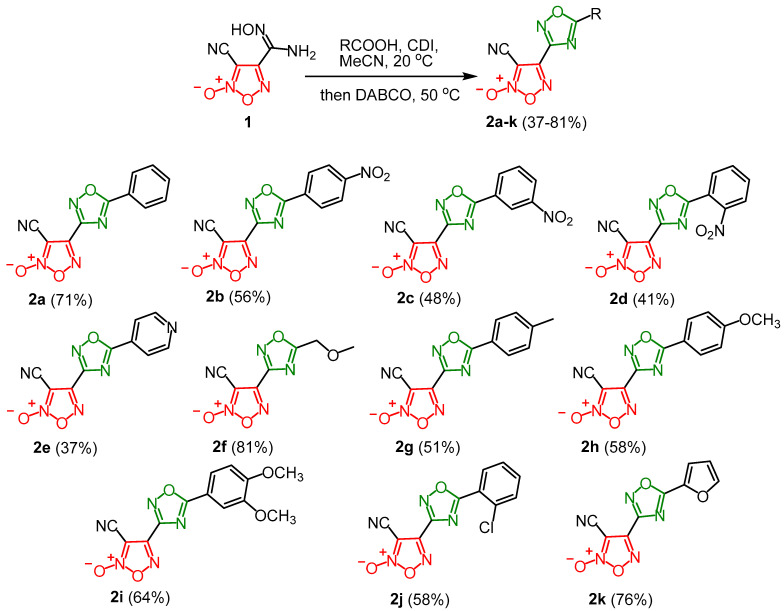
Synthesis of (1,2,4-oxadiazolyl)furoxans **2a**–**k**. Furoxan fragments are depicted in red color; 1,2,4-oxadiazole fragments are depicted in green color.

Having established the optimized conditions, we proceeded to explore the substrate scope of this reaction. In general, amidoxime **1** yielded the desired biheterocyclic compounds in good-to-high yields. In comparison with **2a,** which was isolated in a 71% yield, nitrophenyl-substituted derivatives **2c–d**, as well as pyridyl-derived 1,2,4-oxadiazole **2e**, were isolated in somewhat lower yields ranging from 37% to 48%. On the contrary, nitrophenyl-substituted derivative **2b** and biheterocycles **2f–j** incorporating electron-donating aliphatic or aromatic moieties were obtained in better yields (up to 81%). Furyl-substituted derivative **2k** was also isolated in a good 76% yield (Scheme 1).

**Scheme 2:**
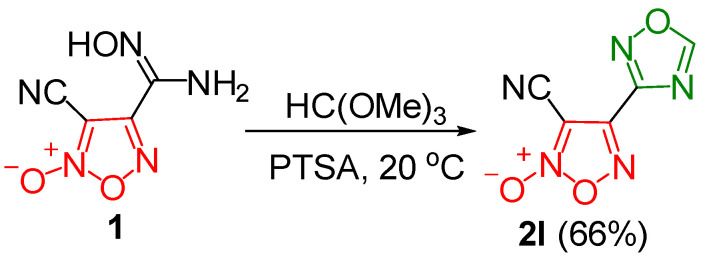
Synthesis of (1,2,4-oxadiazolyl)furoxan **2l**.

In addition, we synthesized unsubstituted 3-cyano-4-(1,2,4-oxadiazolyl)furoxan **2l** via the reaction of furoxanylamidoxime **1** with trimethyl orthoformate under acid catalysis (either *p*-toluenesulfonic acid (PTSA) or BF_3_·OEt_2_). Compound **2l** was isolated in a good yield (66%) (Scheme 2).

All synthesized compounds underwent comprehensive characterization using IR, ^1^H, and ^13^C nuclear magnetic resonance (NMR) spectroscopy (Figures S1.1–S1.24) and high-resolution mass spectrometry (HRMS). For example, in compounds **2a**–**l**, the chemical shift in the CN group in the ^13^C NMR spectrum at the furoxan ring’s (C3) position was observed around 97.0–98.3 ppm, while the chemical shifts for C3 and C4 of the furoxan ring ranged between 105.1 and 107.2 ppm, and between 146.0 and 151.9 ppm, respectively.

### 3.2. X-Ray Crystallography and DFT Calculations

**Figure 2:**
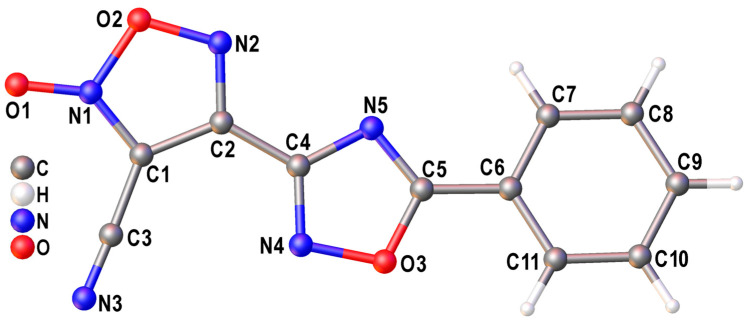
Molecular structure of **2a** according to the single-crystal X-ray diffraction study. Non-hydrogen atoms are given by probability ellipsoids of atomic displacements (*p* = 0.5).

The structure of compound **2a** was confirmed by X-ray diffraction analysis (Figure 2). The molecular conformation of **2a** in a crystal is nearly flat, with C1-C2-C4-N4 and N5-C5-C6-C7 torsion angles equal to 8.1 (2)° and 6.4 (2)°, respectively. According to a search of the Cambridge Structure Database [30], the compounds bear resemblance to previously reported similar structures. For example, the C1-C2-C4-N4 torsion angle in the known substituted (1,2,4-oxadiazolyl)furoxans and (1,2,4-oxadiazolyl)furazans equals, on average, 12.1°, with a mean deviation of 10.9° (85 molecules from 74 analyzed crystal structures). This agrees well with the density functional theory (DFT) calculations of the equilibrium isolated molecules of **2a** in gas. The root mean square difference for the best overlap of the crystal and simulated gas conformations equals only 0.149 Å for **2a** (Figure S2.1 in the Supplementary Materials). This observation is also valid in terms of energy: at the PBE0-D3/def2TZVP level, the electronic energy difference between crystal and gas conformations is only 2.6 kcal/mol.

**Figure 3:**
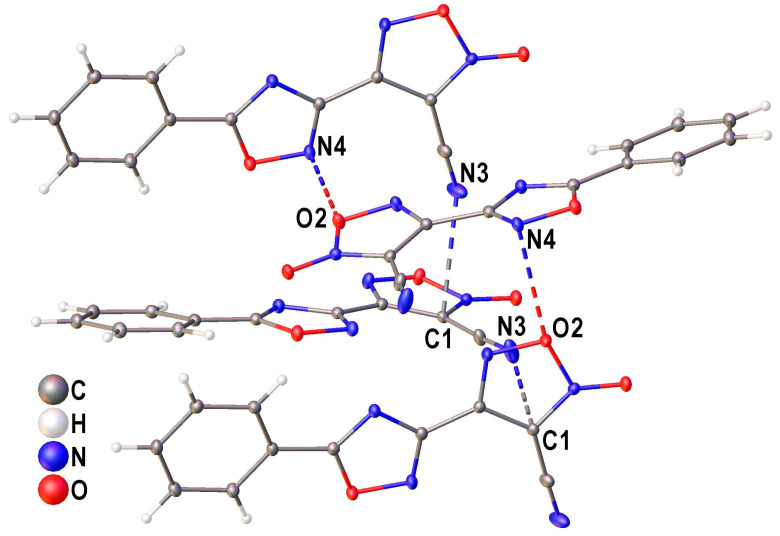
The intralayer organization of molecules in a crystal of **2a**. Shortened contacts corresponding to non-covalent intermolecular interactions are denoted by dashed lines.

The crystal packing motif of **2a** in a crystal features infinite layers of molecules, which are mainly stabilized by the N…π interactions between the nitrile groups and furoxan cycle (N3…C1 2.936(1) Å) and by the O…π interactions between the endocyclic oxygen atoms of the furoxan ring and the 1,2,4-oxadiazolyl fragment (O2…N4 2.931(1) Å, Figure 3). Owing to the directional character of these interactions, the **2a** molecules are arranged almost perpendicular to each other within the layers.

### 3.3. Anticancer Activity

The anticancer properties of **2a**–**l** were tested against a murine mesothelioma cell line (AB1) and a human mesothelioma cell line (JU77) (Table 2 and Figures S3.1–S3.3) in comparison with cisplatin, a clinically approved drug used in combination with pemetrexed in the treatment of MPM [31,32]. Notably, except for **2f**, which showed no cytotoxic effects in both tested cell lines, all the compounds exhibited significant cytotoxicity in both MPM cell lines at low micromolar or sub-micromolar concentrations. The reasons for the absence of cytotoxicity in **2f** remain unclear. However, this leads to the conclusion that the methoxy substituent at the C5 carbon atom of the 1,2,4-oxadiazole ring is not a favorable structural element from a molecular design perspective. The cytotoxicity of **2e** and **2l** was similar to that of cisplatin, while all other tested compounds exhibited enhanced potency in both cell lines. The nitrophenyl-substituted compounds **2b**, **2c**, and **2d** showed increased cytotoxicity compared to the unsubstituted compound **2a**, following this trend: **2a** ≈ **2b** (*para-*) ≤ **2c** (*meta-*) < **2d** (*ortho-*). Notably, **2d** and **2k** (furan substituent) emerged as the most active compounds among all the tested substances in both cell lines. The IC_50_ values for **2d** and **2k** in the AB1 cell line were 1.8 ± 0.2 μM and 1.4 ± 0.4 μM, respectively, and in the JU77 cell line were 0.87 ± 0.07 μM and 0.99 ± 0.13 μM, respectively.

To evaluate the selectivity of the compounds **2a**–**k** towards cancer cells, we assessed their cytotoxicity against non-cancerous MRC-5 human lung fibroblasts. The selectivity factors (SF) determined whether compounds **2a**–**k** preferentially targeted cancer cells (SF > 1) or healthy cells (SF < 1). Interestingly, the less cytotoxic compounds **2l** and **2e** showed low selectivity for cancer cells, suggesting limited therapeutic potential. In contrast, **2c**, **2d**, **2i,** and **2j** exhibited 2.4–3.6 times higher cytotoxicity in human MPM cells compared to human lung fibroblasts. In comparison, previously published hybrids of phenylsulfonyl furoxans and substituted anilinopyrimidines [34] or coumarin [35] demonstrated nanomolar-to-low micromolar cytotoxicity against a panel of cancer cell lines, including lung cancer cell lines. On average, the selectivity of these hybrids towards healthy cells was lower than that of **2c**, **2d**, **2i,** and **2j**. However, these hybrids have not been evaluated in MPM cell lines, making direct comparisons unfeasible.

### 3.4. NO Release

**Figure 4:**
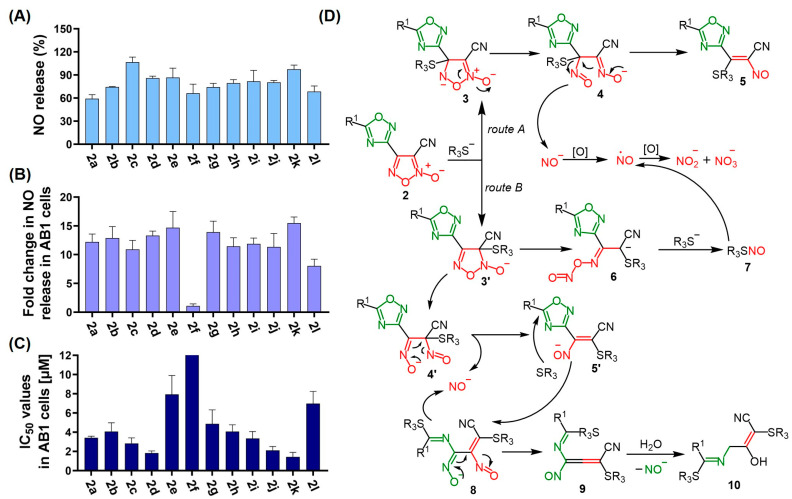
Correlations between NO release and cytotoxicity. (**A**) NO release from compounds **2a**–**l** (100 μM) by the Griess assay after 24 h incubation, as a percentage of NO_2_^−^ concentration relative to the initial concentration of **2a**–**l**. (**B**) NO release from compounds **2a**–**l** (25 μM) in cell lysates from AB1 cells after 24 h incubation, displayed as a fold change of NO_2_^−^ concentration compared to untreated cells. (**C**) IC_50_ values from the MTT assay after 72 h exposure of AB1 cells to compounds **2a**–**l**. (**D**) Proposed mechanism of thiol-dependent NO release. Furoxan fragment and its transformations are depicted in red color; 1,2,4-oxadiazole fragment and its transformations are depicted in green color.

Next, the NO-donor ability of all the synthesized (1,2,4-oxadiazolyl)furoxans **2a**–**l** and furoxanylamidoxime **1** was investigated using the Griess assay after incubation with Griess reagent under physiological conditions at different time intervals (Table 3, Figure 4A). This assay measures the production of nitrite anions resulting from NO oxidation, offering a reliable method for quantifying the released NO content [36]. Two well-known NO-donor compounds, namely, 3-carbamoyl-4-(hydroxymethyl)furoxan (CAS-1609) and 4-ethoxy-3-phenylsulphonylfuroxan (CHF-2363), were used as a reference [36,37]. The NO-releasing capacity was calculated as the NO_2_^−^ concentration relative to the initial concentration of (1,2,4-oxadiazolyl)furoxans **2a**–**l** (100 μM) in terms of percentage. A standard curve was produced by measuring the absorbance changes in various concentrations of sodium nitrite solutions treated with L-cysteine and the Griess reagent system in a phosphate buffer at pH = 7.4.

It was revealed that the NO-donor capacity of (1,2,4-oxadiazolyl)furoxans (42–107%) was significantly higher than that of CAS-1609 (27%). Moreover, for some of the compounds, such as **2c** and **2k**, the concentration of produced NO_2_^−^ exceeded the initial concentrations of the furoxans, leading to values surpassing 100%, indicating the capability of both the furoxan ring and the 1,2,4-oxadiazole scaffold to release NO. This finding aligns with recent literature reports on the NO-donor properties of 1,2,4-oxadiazole derivatives [38,39]. It should be noted that the initial NO release was rapid for all compounds, observed within just 1 h of incubation. In contrast, previously published compounds, such as 4-(2-methylpyridin-5-yloxy)-3-phenylfuroxan and 4-amino-3-(indenotriazin-3-yl)furoxan, only released 10–13% of NO within the same 1 h timeframe [40]. Over the next 24 h, the percentage of NO release increased by 1.2–1.8 times. By 48 h of incubation, the percentage of NO release for the majority of compounds reached a plateau (48 h/24 h = 1.0–1.1). The most effective NO donors were *o*- and *m*-nitrophenyl-substituted **2c,d** and furanyl-substituted **2k** (1,2,4-oxadiazolyl)furoxans, while unsubstituted (1,2,4-oxadiazolyl)furoxan **2l** was the least potent. Subsequently, we conducted an in vitro study to assess NO release in AB1 cells treated with 25 μM of the compounds **2a**–**l** for 24 h (Figure 4B). Overall, all compounds, except **2f**, exhibited comparable levels of NO release. Notably, the sustained NO-releasing ability of furoxans has not been widely investigated. However, a study involving a PEG derivative of phenylfuroxan and its micelle formation revealed that the NO release could last for up to 3 d [41]. The NO-releasing activity of this compound dramatically increased between 1 and 3 d, with the NO release reaching a plateau around 2 d, mirroring the behavior observed for compounds **2a**–**l** in our experiments.

The comparison of the cytotoxicity of compounds **2a**–**l** in AB1 cells after 72 h of exposure (Figure 4C) with the NO release after 24 h (Figure 4A) revealed certain correlations between these parameters. For instance, compounds **2e** and **2f**, which exhibited the lowest anticancer activity, also showed less efficient NO release. Notably, compounds **2c** and **2k**, which exhibited the highest NO release from both heterocycles, were among the most cytotoxic compounds. These findings suggest that the potent antiproliferative activities of (1,2,4-oxadiazolyl)furoxans **2a**–**l** may be partially linked to NO release. However, other factors, like potential variations in intracellular accumulation, should also be considered.

According to the literature [42], we have proposed a mechanism of thiol-dependent NO release from heterocyclic assemblies **2a**–**l** (Figure 4D). The furoxan ring’s strong electron-withdrawing nature enhances the electrophilicity of both carbon atoms within the heterocycle, rendering them reactive towards nucleophilic species. Consequently, two primary pathways for NO release from furoxans emerge, contingent on whether the thiolate anion, derived from a thiol-containing molecule such as cysteine, interacts with the C3 or C4 carbon atom of the furoxan ring. Routes A and B exhibit similarities, starting with the formation of intermediates **3** and **3′**, followed by endocyclic cleavage of the N-O bond, yielding anions **4** and **4′**. Intermediates **4** and **4′** then decompose into isomeric nitrosoethylenes **5** and **5′**, with elimination of the nitroxyl anion. These species undergo oxidation to NO, which may subsequently oxidize to nitrite or nitrate. The main difference between routes A and B arises from the potential cleavage of the C–N bond in the intermediate **3′** in the latter mechanism, potentially forming nitroso-oxime **6**. This, in turn, results in the nitrosation of the thiolate anion, leading to the formation of *S*-nitrosothiol **7** and its subsequent decomposition into NO and to the regeneration of the thiol. However, based on previous research [38,39] and data from NO-donor studies of compounds **2a**–**l**, it is possible that 1,2,4-oxadiazoles may undergo cleavage upon the attack of a thiolate anion at the C5 atom in the 1,2,4-oxadiazole ring. This scenario would give rise to an anion **8**, which subsequently decomposes into an allene-type intermediate **9** with the release of a nitroxyl anion. This unstable intermediate then hydrolyzes to form hydroxy sulfinyl nitrile **10**. This proposed mechanism potentially explains why compound **2k** exhibits a NO-donating capacity of more than 100%, implying that the compound undergoes a series of reactions that result in the two oxadiazolyl cycles’ release of NO.

## 4. Conclusions

**In this study** we established a straightforward synthetic route for the preparation of (1,2,4-oxadiazolyl)furoxans. This method involved the reaction of furoxanylamidoximes with carboxylic acids, eliminating the need to isolate unstable intermediates. As a result, 11 novel (1,2,4-oxadiazolyl)furoxans **2a**–**k** have been produced in moderate-to-good yields and fully characterized by spectrochemical methods. The structure of compound **2a** was confirmed by X-ray diffraction analysis. In addition, we synthesized an unsubstituted 1,2,4-oxadiazole hybrid **2l** via the reaction of furoxanylamidoxime **1** with trimethyl orthoformate under acid catalysis. The anticancer properties of compounds **2a**–**l** were evaluated against a murine mesothelioma cell line (AB1) and a human mesothelioma cell line (JU77), in comparison with cisplatin. The nitrophenyl-substituted compound **2d** and furan-substituted compound **2k** emerged as the most active compounds among all the tested substances in both cell lines, displaying IC_50_ values ranging between 0.87 and 1.8 μM. In contrast, the unsubstituted analog **2l** was at least 3 times less cytotoxic (5.4–7.0 μM), underscoring the impact of the substituents on the 1,2,4-oxadiazole ring. Furthermore, similar trends were observed with respect to the compounds’ selectivity toward cancer cells. For example, compound **2d** exhibited 2.4–3.6 times higher cytotoxicity in MPM cells compared to human lung fibroblasts, whereas the unsubstituted compound **2l** did not show any preference for cancer cells. The anticancer properties of the novel oxadiazole assemblies were, to some extent, connected to their ability to donate NO. We have proposed a mechanism detailing the thiol-dependent release of NO from the heterocyclic assemblies **2a**–**l**, offering insights into the observed NO release profiles. It is important to note that the incorporation of the additional NO-donor 1,2,4-oxadiazolyl fragment significantly increased the NO-releasing capability of the furoxan hybrids when compared to previously reported furoxans lacking 1,2,4-oxadiazoles. However, it is essential to consider potential differences in intracellular accumulation and other factors unrelated to NO donation that might contribute to their biological activity. In conclusion, the results suggest that (1,2,4-oxadiazolyl)furoxans hold promise as a class of NO-donating prodrugs for MPM. Future research directions will involve exploring bis(1,2,4-oxadiazolyl)furoxans and assessing the impact of incorporating a second 1,2,4-oxadiazolyl fragment on the anticancer efficacy of these oxadiazole assemblies.

## Figures and Tables

**Table 1:** Optimization of the reaction conditions for the synthesis of **2a**.

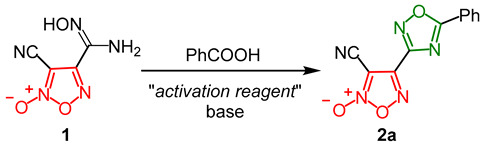					
Entry	Activation Reagent	Base	Time, h	T, °C	Yield of 2a, *^a^* %
1	SOCl_2_	Cs_2_CO_3_	10	20	20
2	SOCl_2_	Cs_2_CO_3_	6	20	14
3	SOCl_2_	Cs_2_CO_3_	12	20	48
4	SOCl_2_	Cs_2_CO_3_	5	50	21
5	SOCl_2_	Cs_2_CO_3_	48	20	23
6	SOCl_2_	TEA	7	20	33
7	CDI	DBU	12	20	37
8	CDI	TEA	7	20	52
9	CDI	DABCO	3	20	56
10	CDI	DABCO	3	50	71

*^a^* Isolated yields.

**Table 2:** Cytotoxicity of (1,2,4-oxadiazolyl)furoxan **2a**–**l** in comparison with cisplatin.

Compound	IC50, μM ^a^			SF_JU77_^b^
	AB1	JU77	MRC-5	
**2a**	3.4 ± 0.2	3.7 ± 1.0	3.7 ± 0	1.0
**2b**	4.1 ± 0.9	2.7 ± 0.2	4.4 ± 0.6	1.6
**2c**	2.8 ± 0.5	1.2 ± 0.2	4.3 ± 0.3	3.6
**2d**	1.8 ± 0.2	0.87 ± 0.07	2.2 ± 0.2	2.5
**2e**	7.9 ± 2.0	4.2 ± 0.8	4.7 ± 0.3	1.1
**2f**	>150	>150	>150	-
**2g**	4.9 ± 1.4	3.1 ± 0.2	4.8 ± 0.3	1.5
**2h**	4.1 ± 0.7	2.9 ± 0.3	4.2 ± 0.7	1.4
**2i**	3.3 ± 0.7	1.6 ± 0.4	4 ± 0.6	2.5
**2j**	2.1 ± 0.3	1.4 ± 0.2	3.3 ± 0.3	2.4
**2k**	1.4 ± 0.4	0.99 ± 0.13	1.5 ± 0.3	1.5
**2l**	7.0 ± 1.3	5.4 ± 1.3	4.6 ± 1	0.9
**Cisplatin ^c^**	5.8 ± 1	4.2 ± 0.5	3.1 ± 1.2	0.7

^a^ 50% effective concentrations IC_50_ were determined in AB1 (mouse malignant mesothelioma), JU77 (human malignant mesothelioma), and MRC-5 (human embryonic lung fibroblast) cell lines using the MTT assay with a 72 h exposure time. Mean standard deviations were calculated based on at least three independent experiments. ^b^ The selectivity factors (SFs) were determined as IC_50_(MRC-5)/IC_50_(JU77). ^c^ The values are taken from the ref. [33].

**Table 3:** NO-donor ability of (1,2,4-oxadiazolyl)furoxans **2a**–**l**.

Compound	NO Released (%) ^a^			Fold Change		
	1 h	24 h	48 h	24 h/1 h	48 h/1 h	48 h/24 h
**1**	70 ± 5	81 ± 4	84 ± 10	1.2	1.2	1.0
**2a**	50 ± 8	59 ± 5	89 ± 6	1.2	1.8	1.5
**2b**	51 ± 11	74 ± 1	80 ± 4	1.5	1.6	1.1
**2c**	61 ± 8	107 ± 8	107 ± 10	1.8	1.8	1.0
**2d**	69 ± 1	86 ± 3	89 ± 3	1.2	1.3	1.0
**2e**	53 ± 7	87 ± 13	87 ± 11	1.6	1.6	1.0
**2f**	50 ± 7	66 ± 11	71 ± 11	1.3	1.4	1.1
**2g**	59 ± 1	74 ± 6	81 ± 10	1.3	1.4	1.1
**2h**	54 ± 6	79 ± 5	86 ± 4	1.5	1.6	1.1
**2i**	54 ± 14	82 ± 14	94 ±14	1.5	1.7	1.1
**2j**	53 ± 4	80 ± 3	84 ± 2	1.5	1.6	1.1
**2k**	73 ± 7	97 ± 6	104 ± 12	1.3	1.4	1.1
**2l**	42 ± 5	68 ± 8	72 ± 8	1.6	1.7	1.1
**CAS-1609**	27 ± 3	68 ± 6	73 ± 6	2.5	2.7	1.1
**CHF-2363**	26 ± 10	71 ± 4	77 ± 9	2.7	3.0	1.1

^a^ Expressed as % NO_2_^−^ (μmol/μmol).

## Data Availability

The original contributions presented in this study are included in the article. Further inquiries can be directed to the corresponding authors.

## Supplementary Materials

The following supporting information can be downloaded at https://www.mdpi.com/article/10.3390/pharmaceutics17020230/s1, Figure S1.1–Figure S1.24: ^1^H NMR spectra; Table S2.1. Main crystallography data and refinement details for the **2a** structure, Figure S2.1: the best root mean square of crystal and gas conformations of **2a** crystallographic data, and Figure S3.1–Figure S3.3: Concentration-effect curves for **2a-l** and **3a-l** in AB1, JU77 and MRC-5 cell lines upon 72 h exposure.
