# Supplementary material for: Exploring the Anticancer Potential of NO-Donor Oxadiazole Assemblies Against Malignant Pleural Mesothelioma
Source: Pharmaceutics. 2025 Feb 10;17(2):230. doi: 10.3390/pharmaceutics17020230 (PMC11859074; doi:10.3390/pharmaceutics17020230)
Supplement: Supplementary file 1 [file pharmaceutics-17-00230-s001.zip › pharmaceutics-3422932-supplementary.pdf]

Table of Contents

S1. NMR spectra **2**

S2. Crystallographic data **26**

S3. Biological activity **278**

S1. NMR spectra

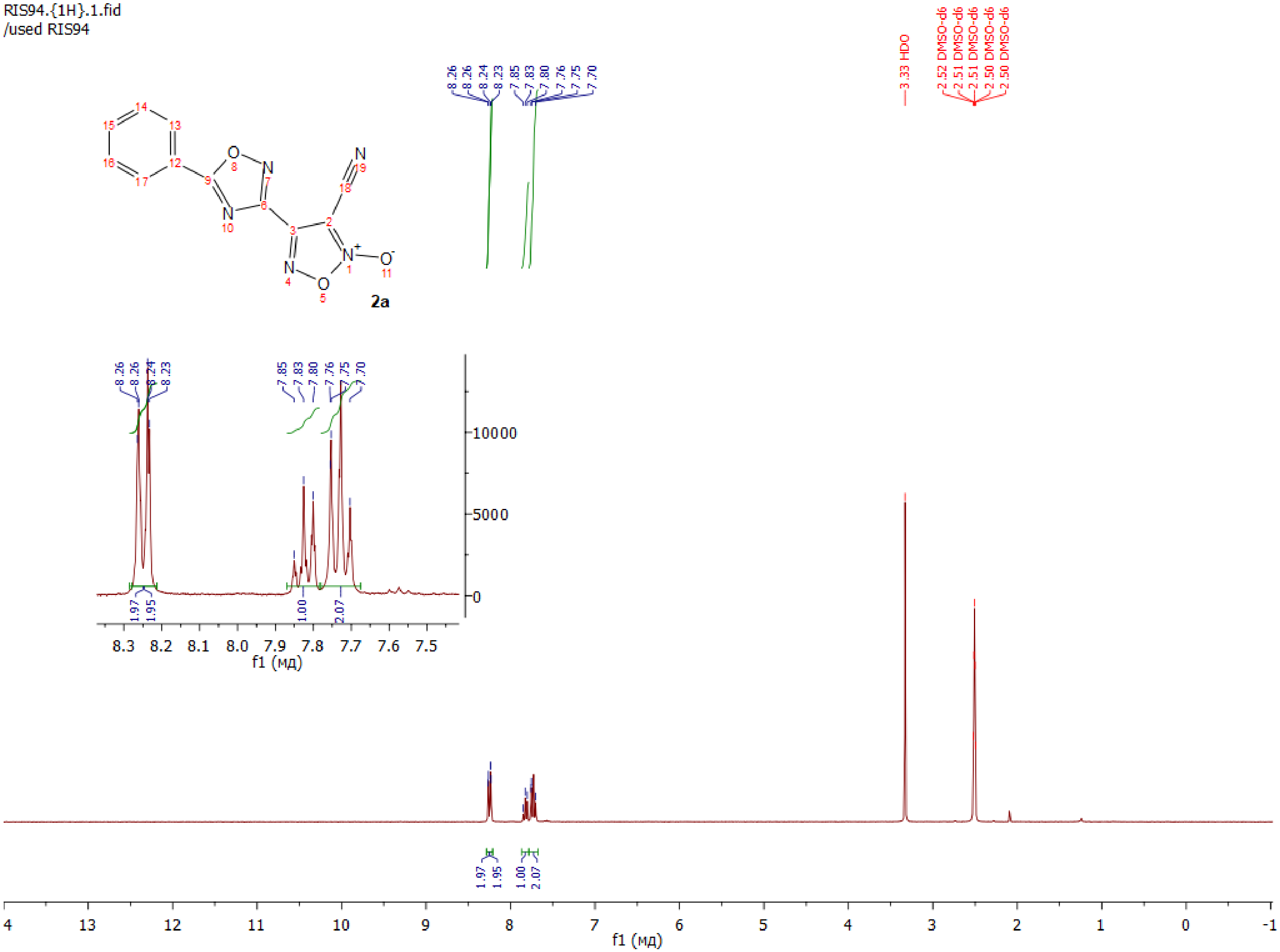

Figure S1.1. ^{1}H NMR spectrum of **2a**, DMSO-[d_6]

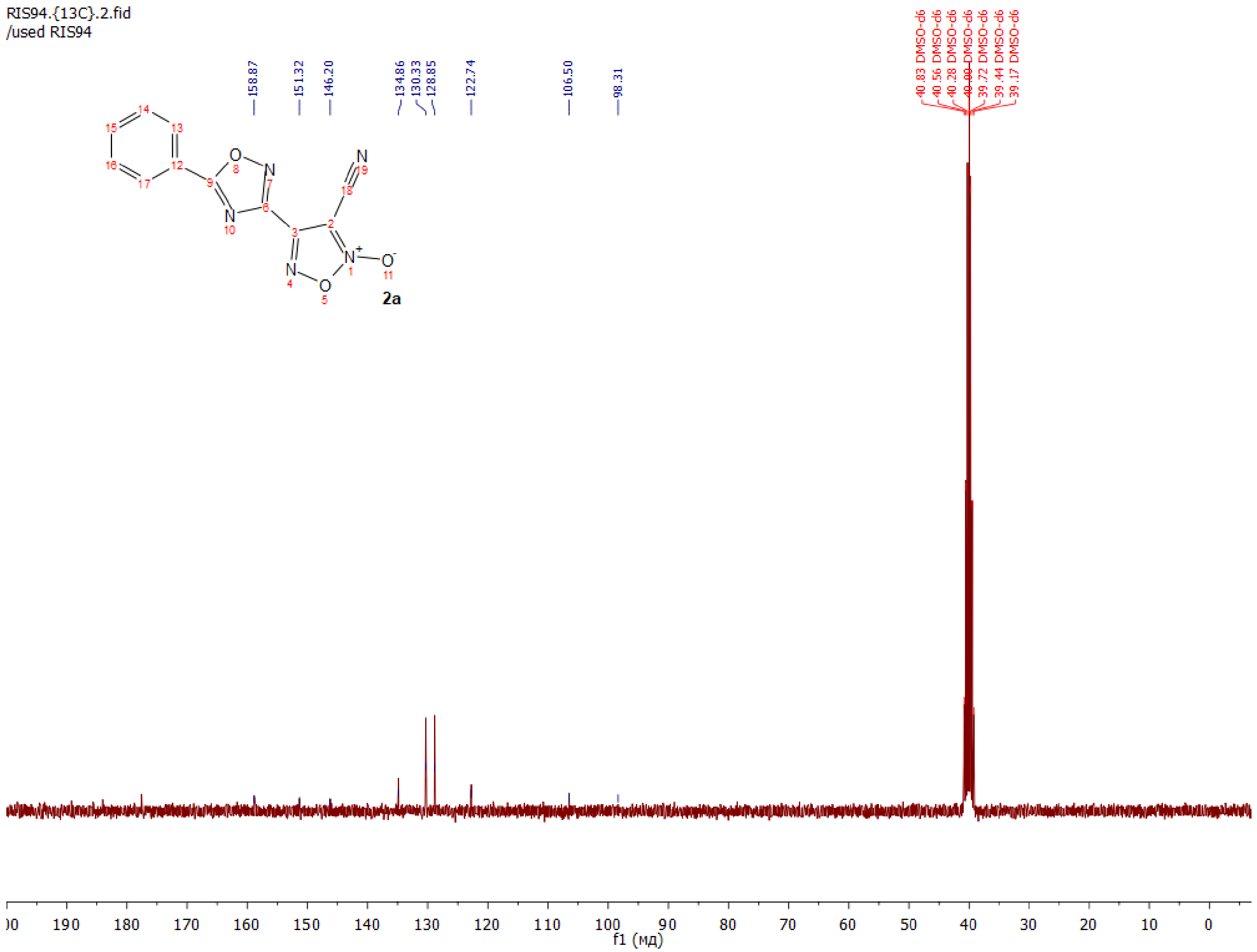

Figure S1.2. ^{13}C NMR spectrum of **2a**, DMSO-[d$_6$]

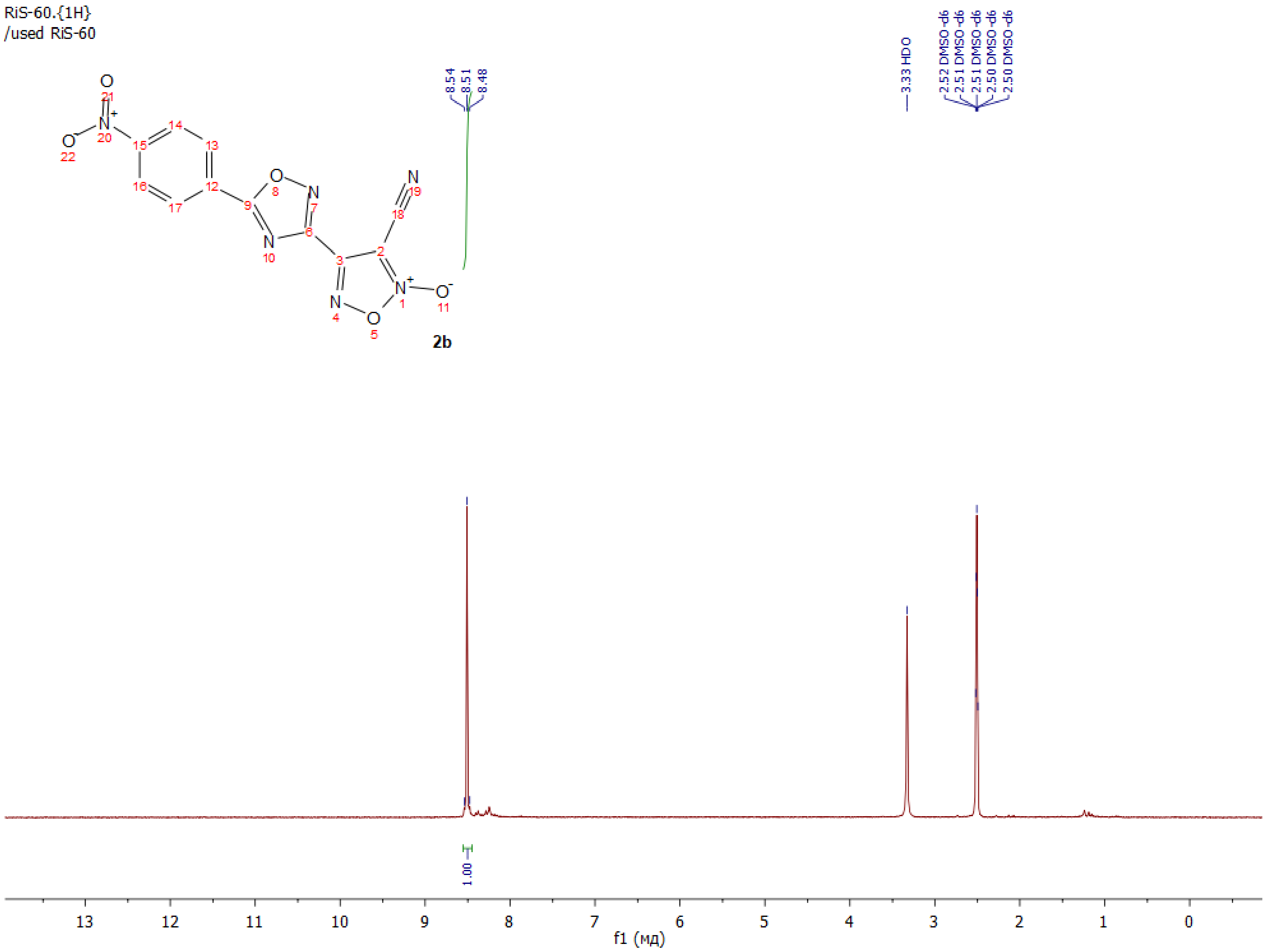

Figure S1.3. ^{1}H NMR spectrum of **2b**, DMSO-[d_6]

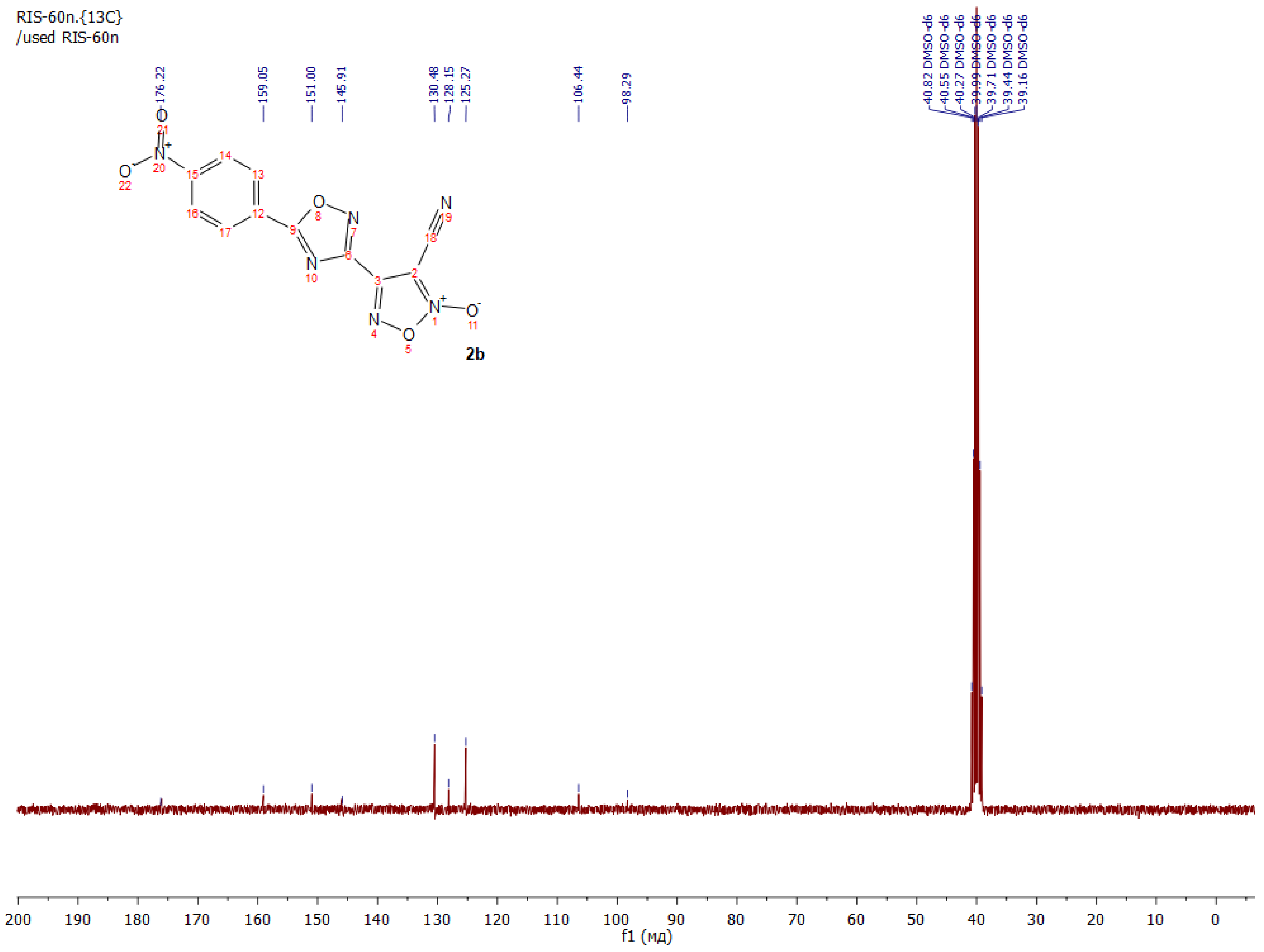

Figure S1.4. ^{13}C NMR spectrum of **2b**, DMSO-[d_6]

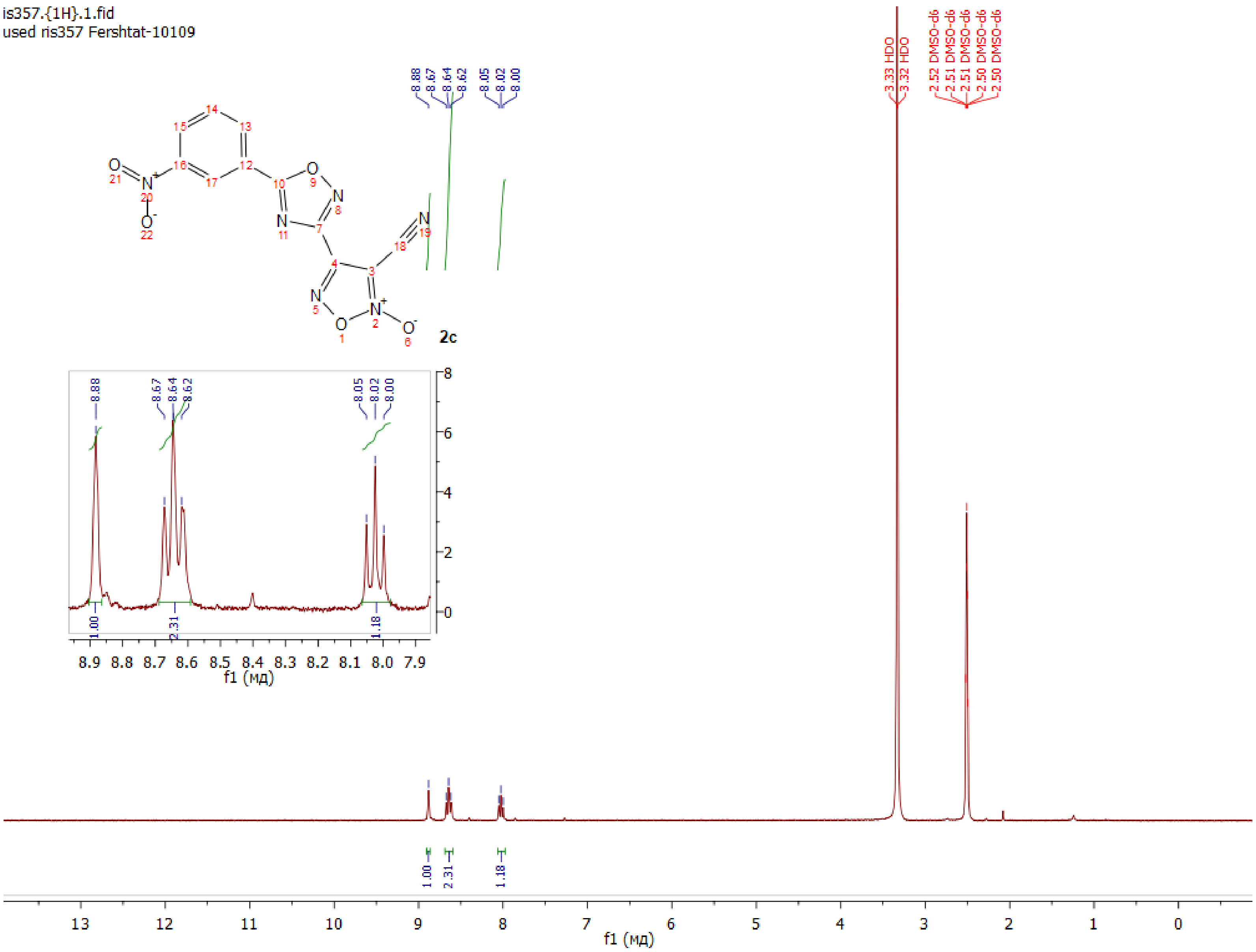

Figure S1.5. ^{1}H NMR spectrum of **2c**, DMSO-[d$_6$]

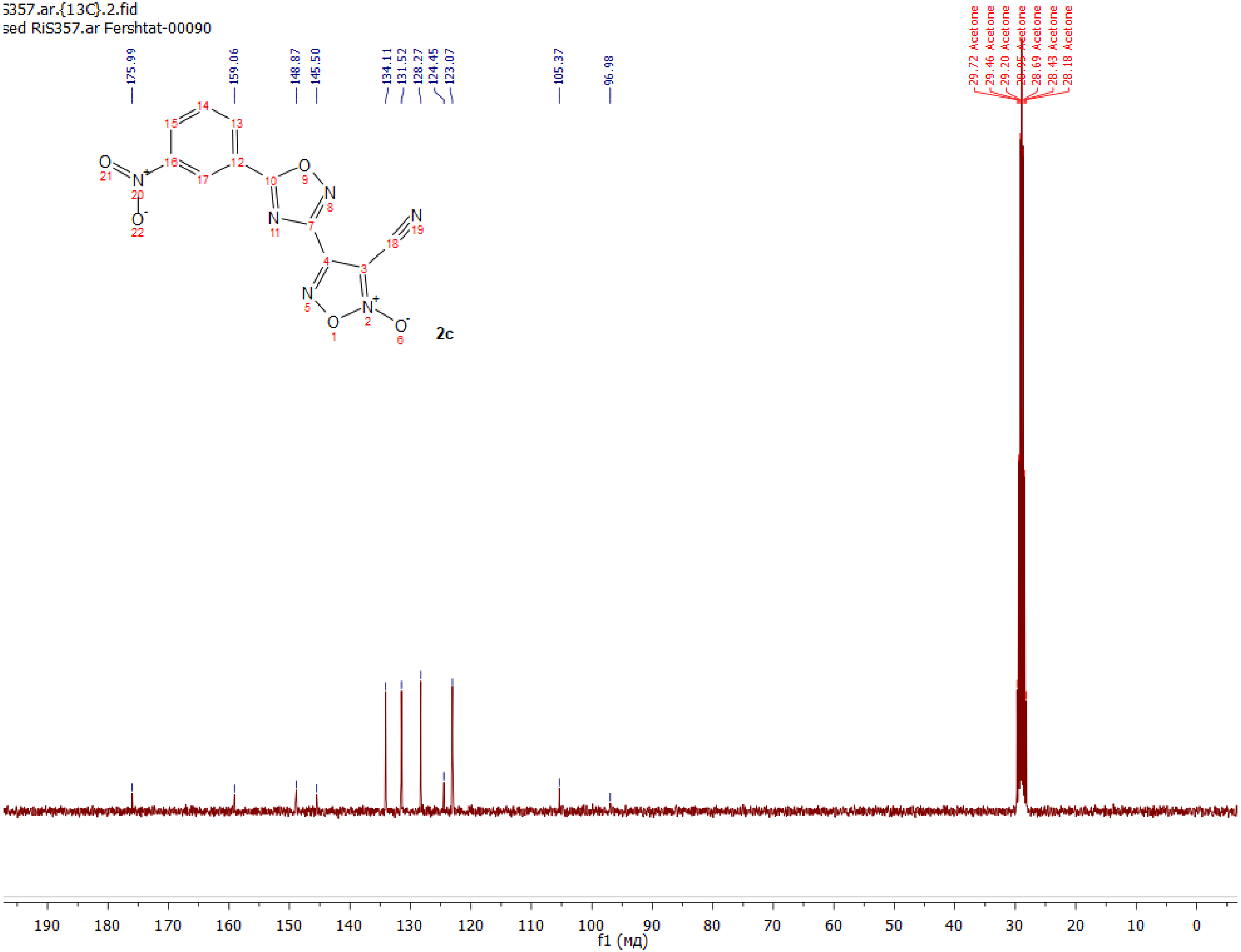

Figure S1.6. ^{13}C NMR spectrum of **2c**, Acetone-[d$_6$]

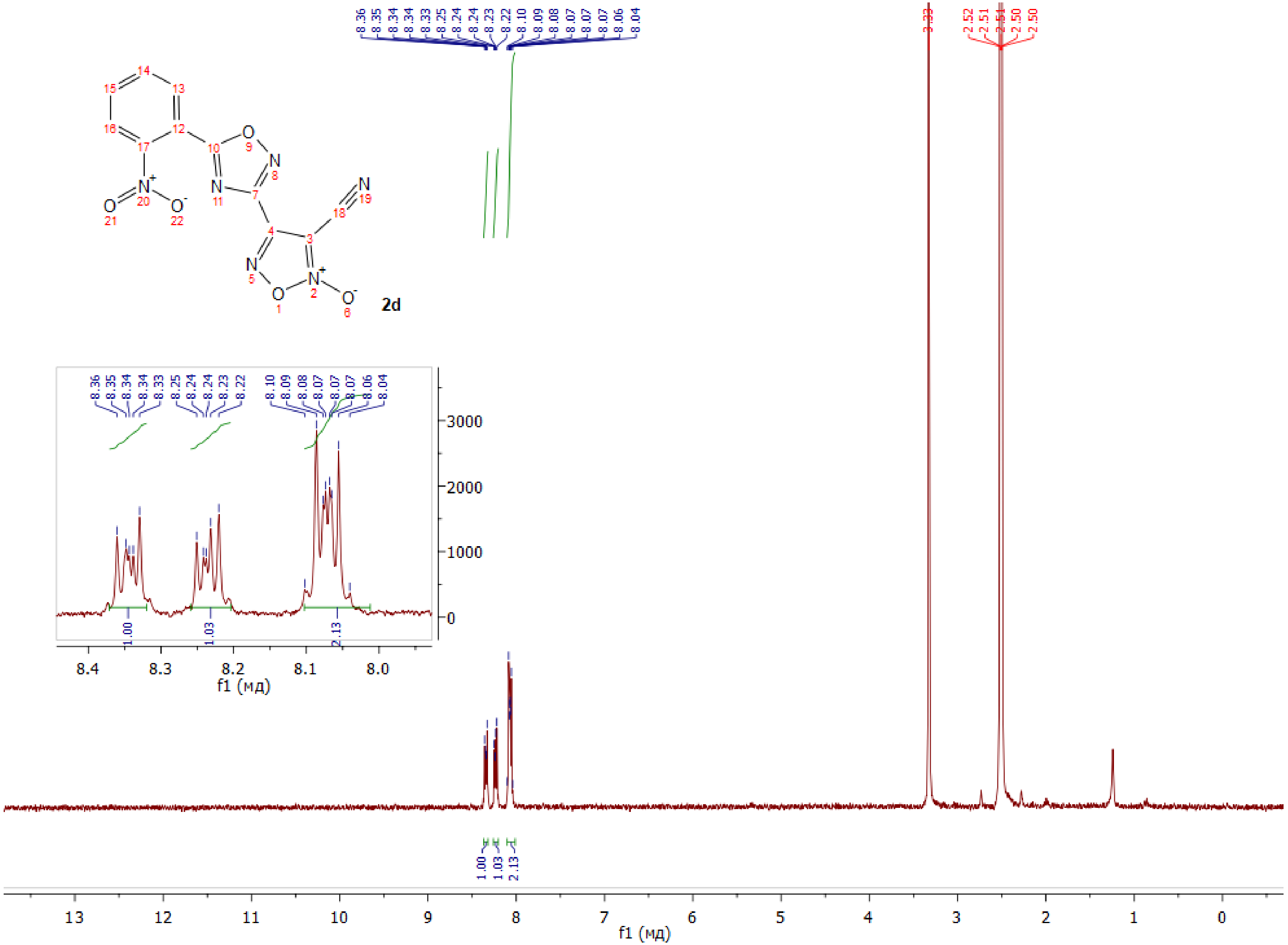

Figure S1.7. ^{1}H NMR spectrum of **2d**, DMSO-[d_6]

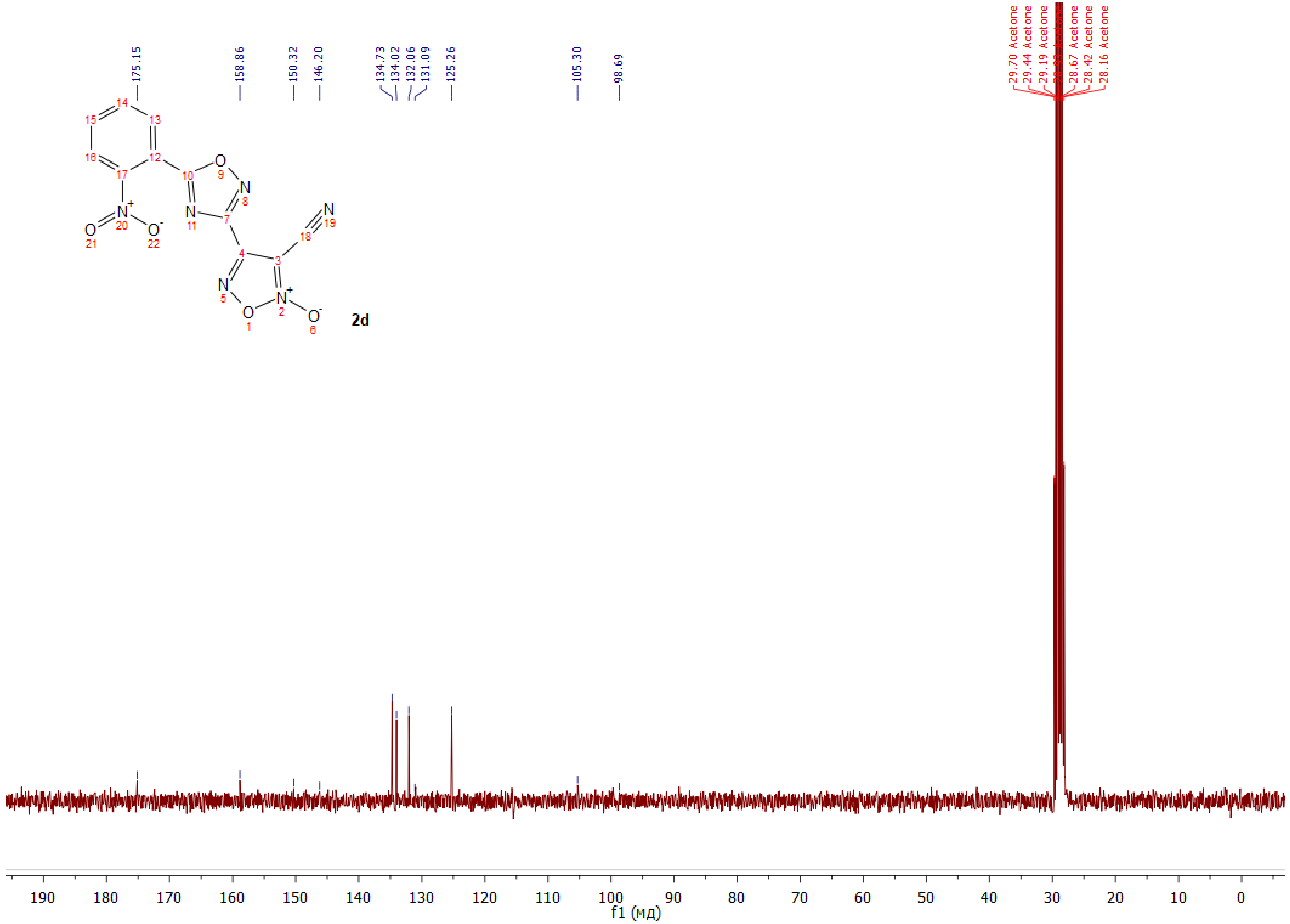

Figure S1.8. ^{13}C NMR spectrum of **2d**, Acetone-[d_6]

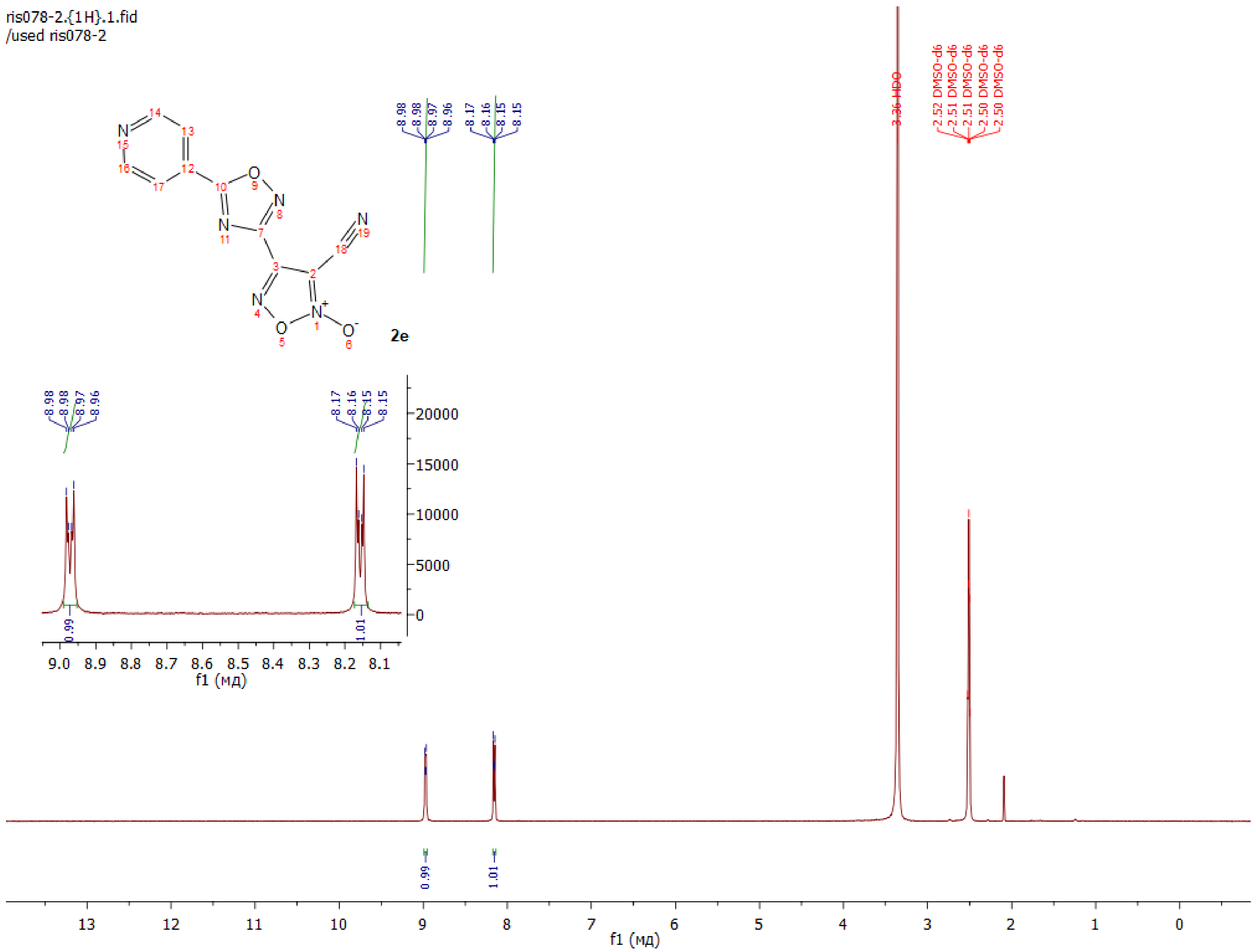

Figure S1.9. ^{1}H NMR spectrum of **2e**, DMSO-[d_6]

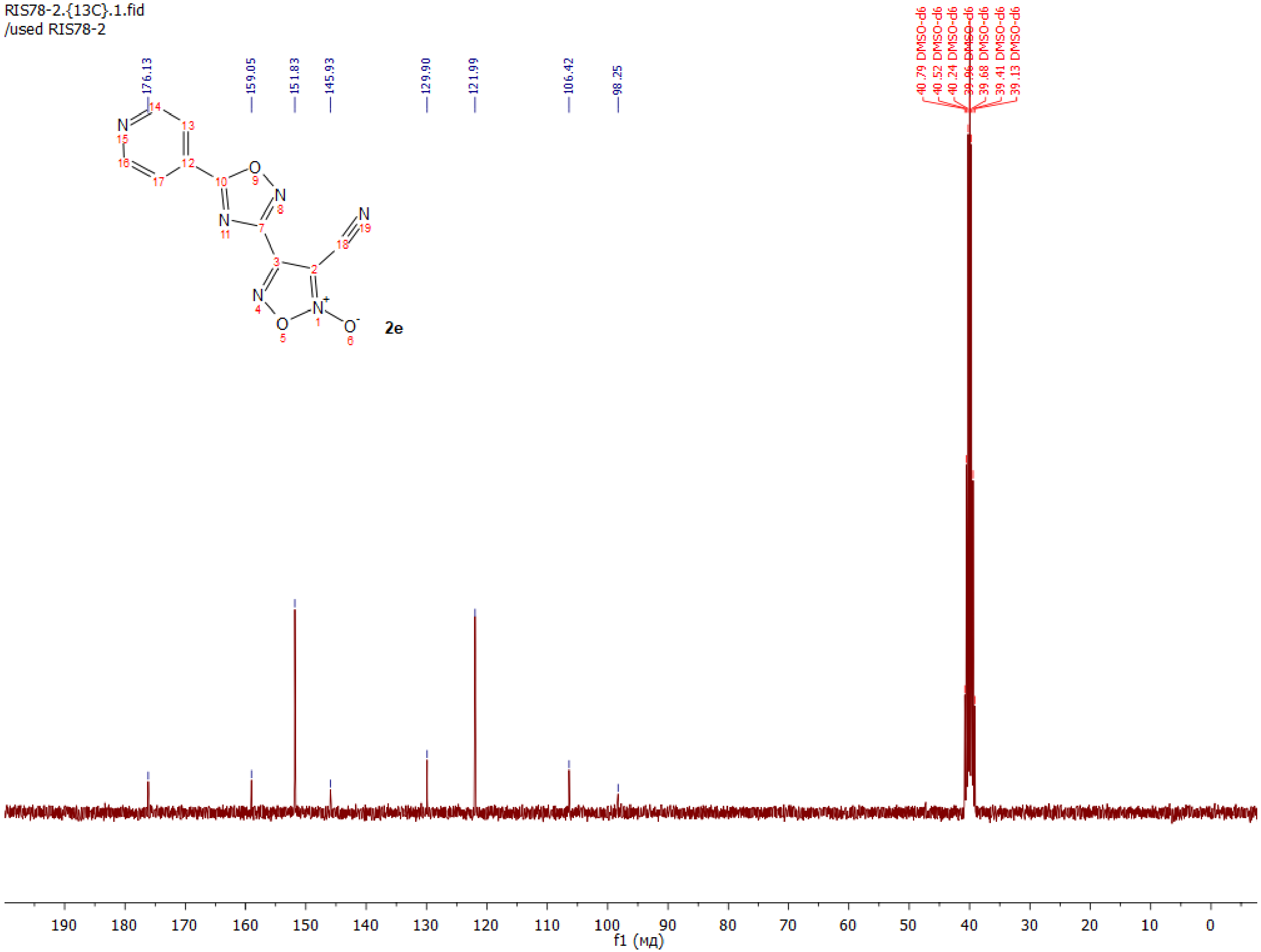

Figure S1.10. ^{13}C NMR spectrum of **2e**, DMSO-[d$_6$]

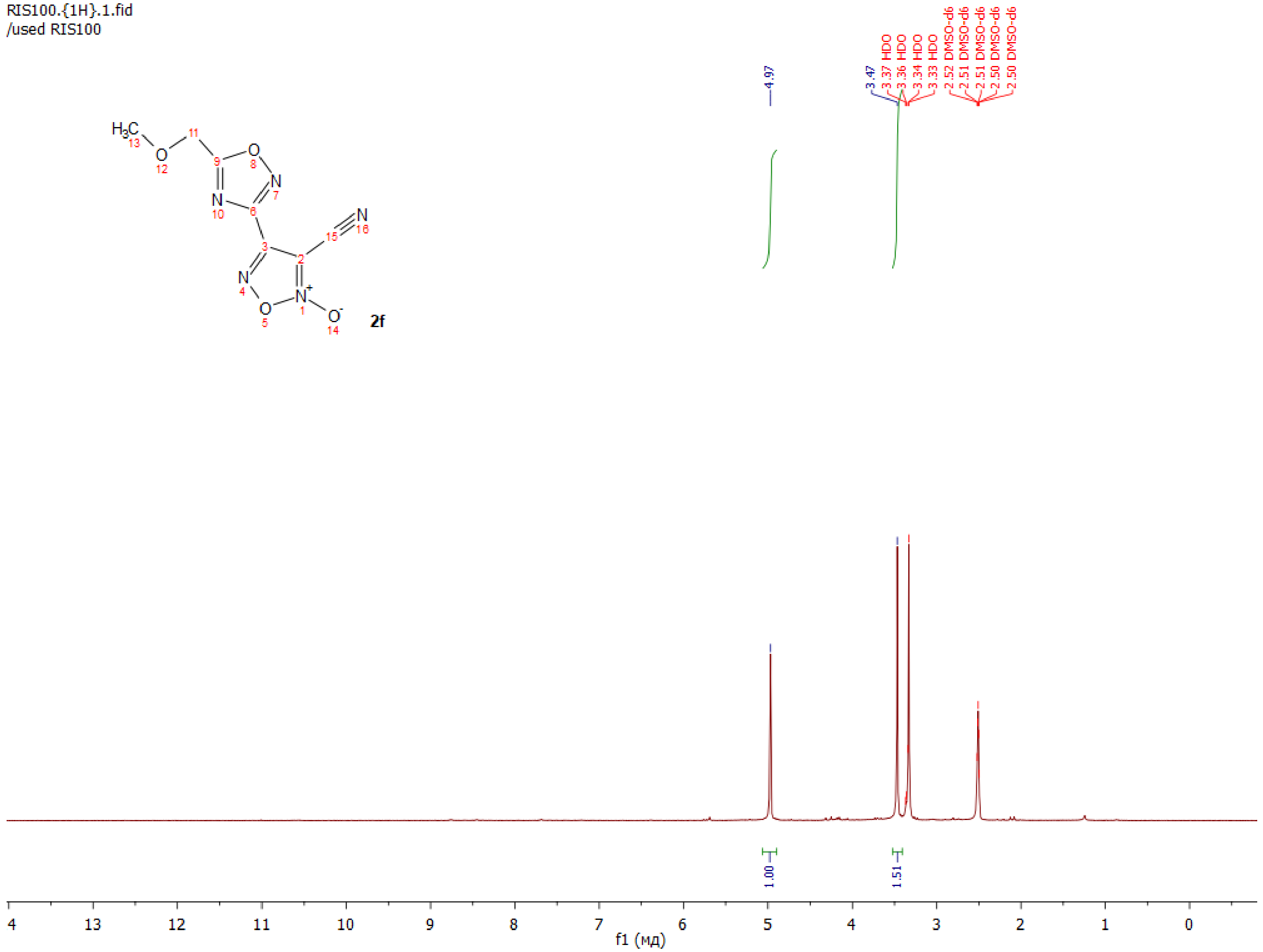

Figure S1.11. ^{1}H NMR spectrum of **2f**, DMSO-[d_6]

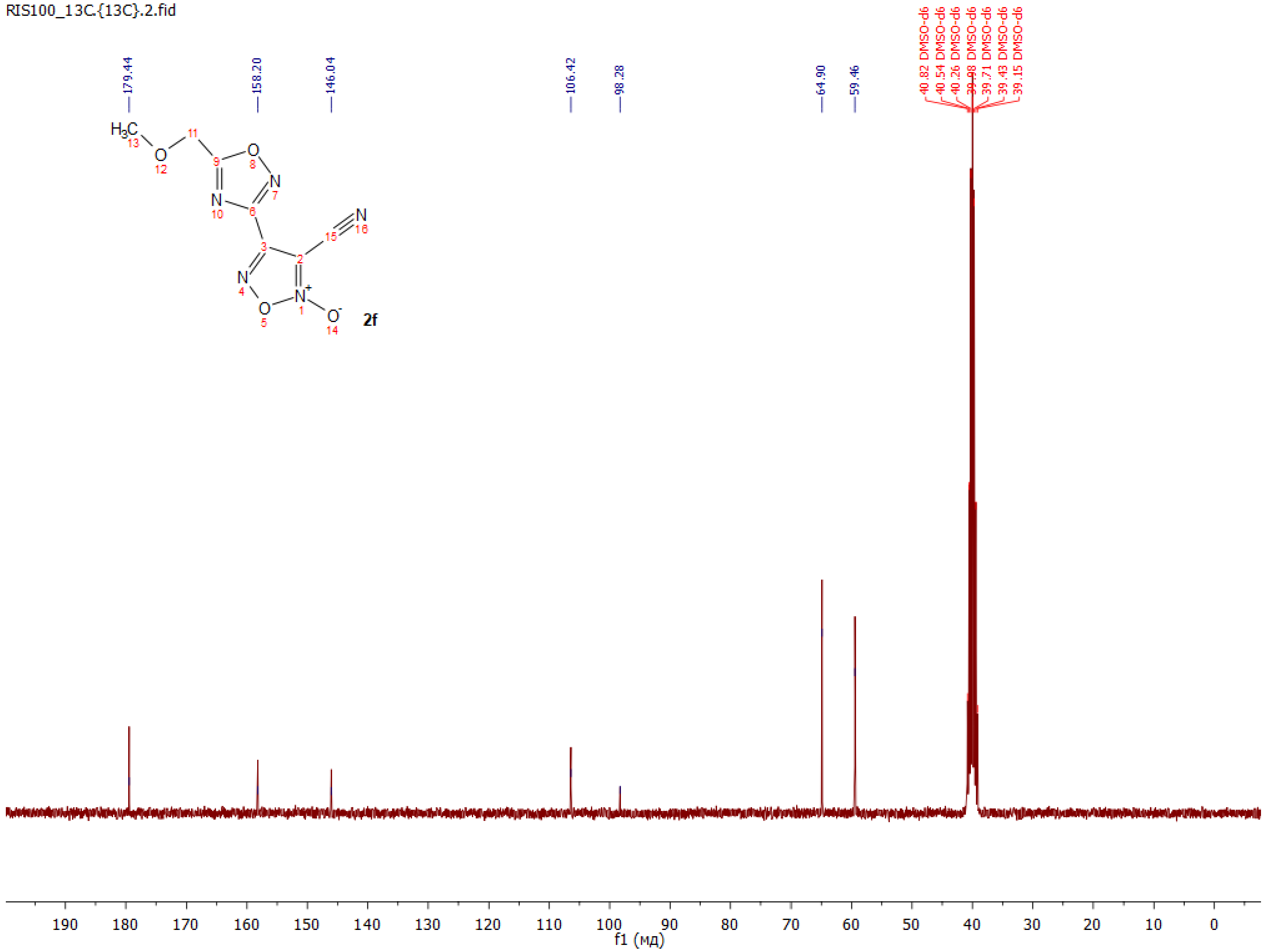

Figure S1.12. ^{13}C NMR spectrum of **2f**, DMSO-[d_6]

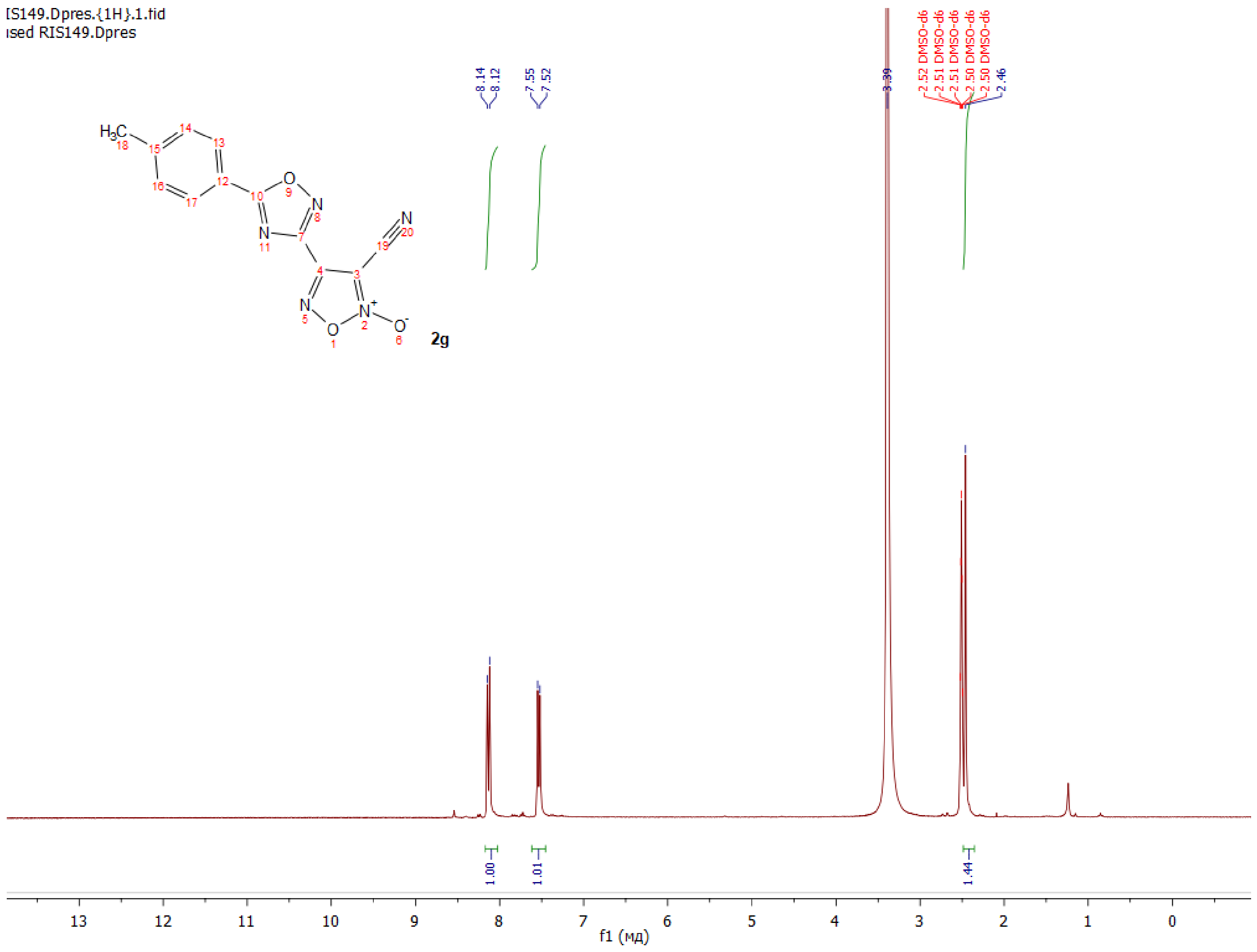

Figure S1.13. ^{1}H NMR spectrum of **2g**, DMSO-[d$_6$]

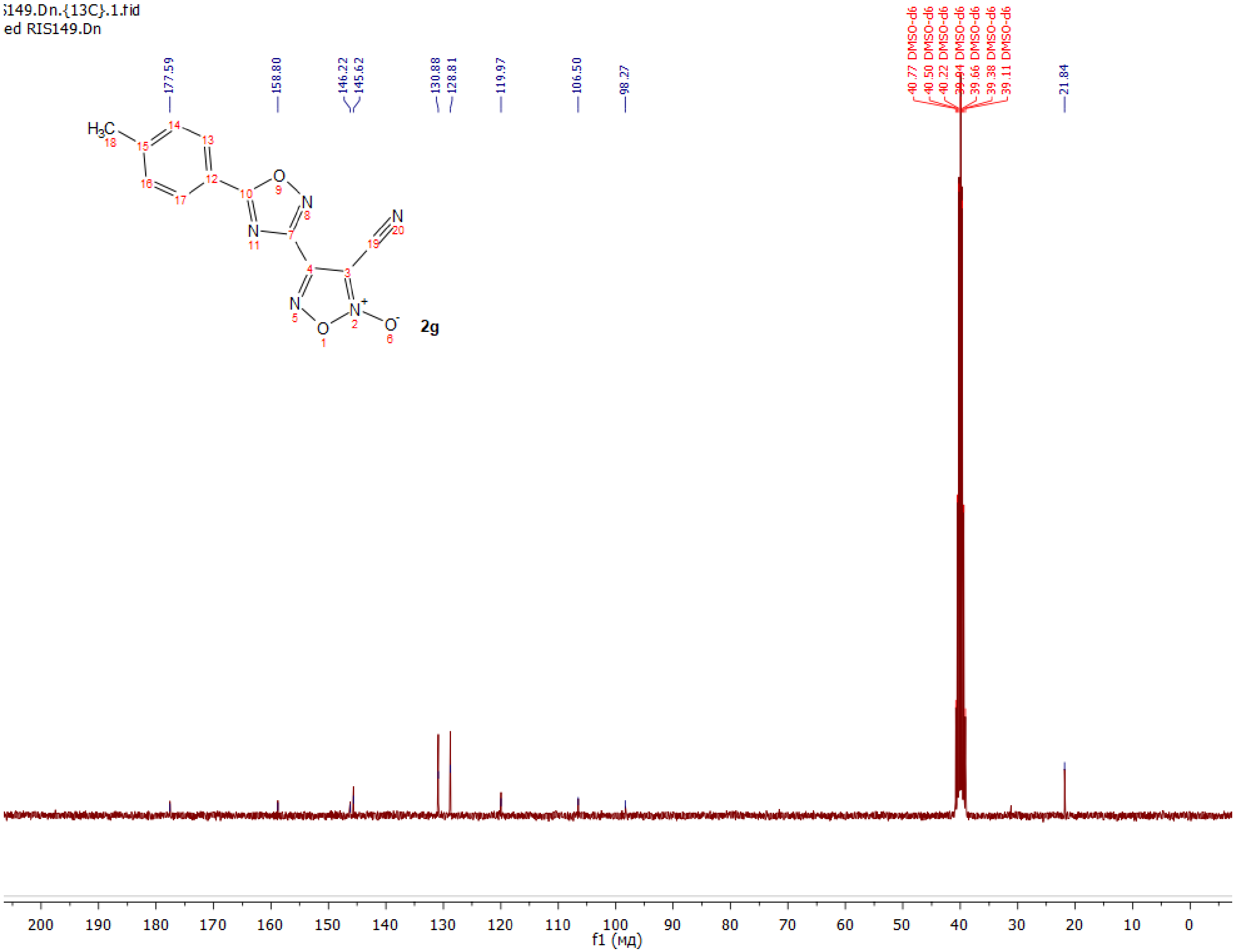

Figure S1.14. ^{13}C NMR spectrum of **2g**, DMSO-[d_6]

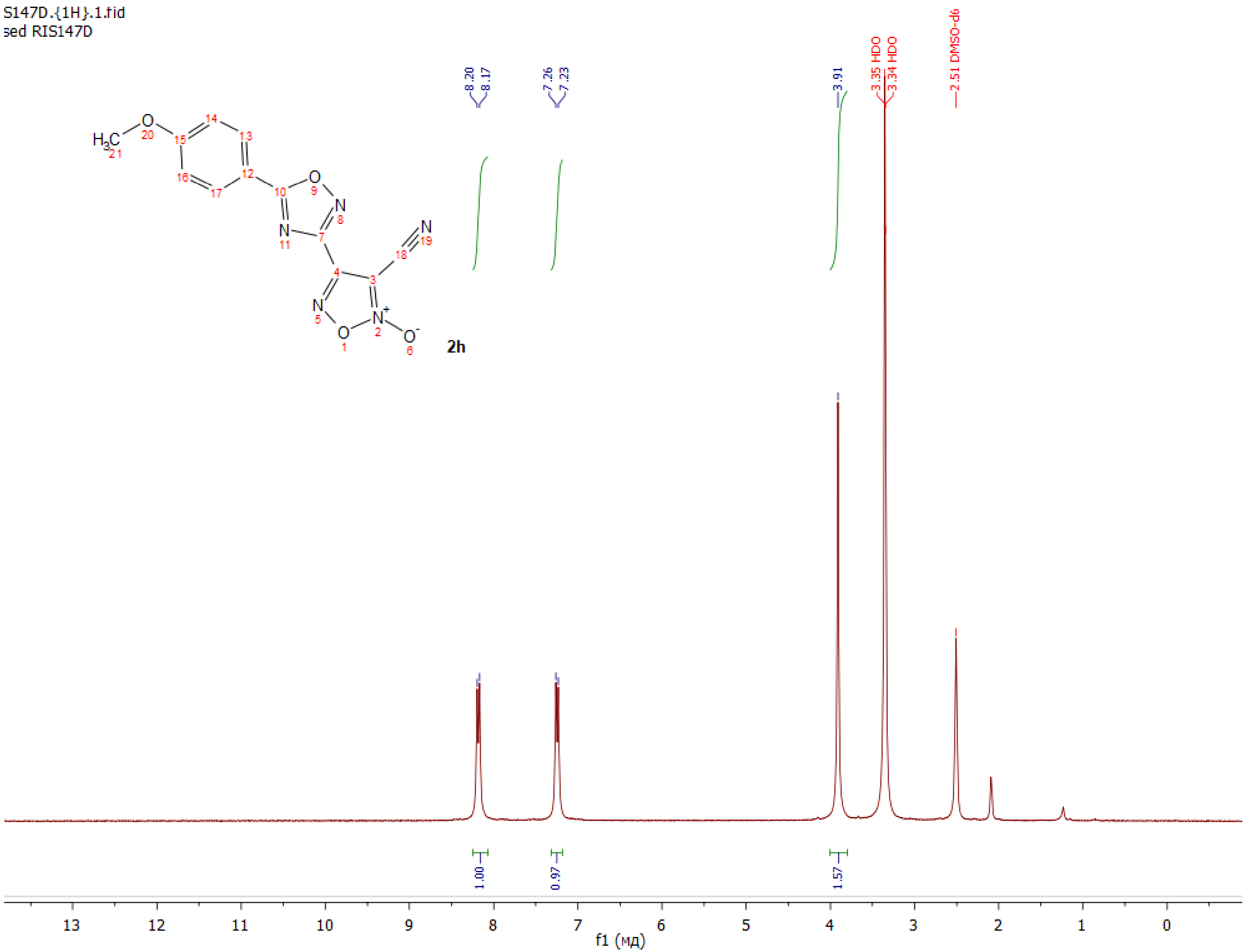

Figure S1.15. ^{1}H NMR spectrum of **2h**, DMSO-[d_6]

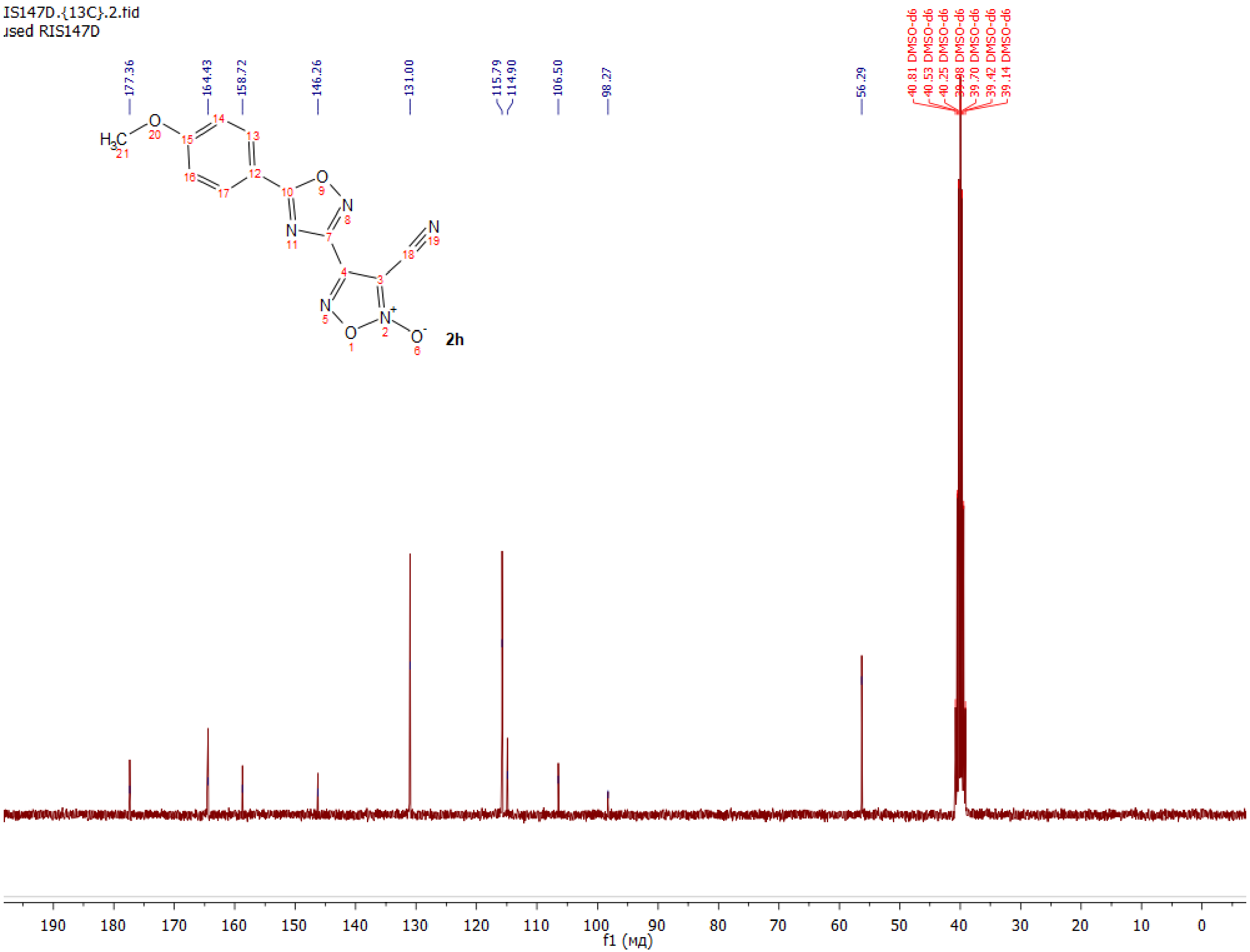

Figure S1.16. ^{13}C NMR spectrum of **2h**, DMSO-[d_6]

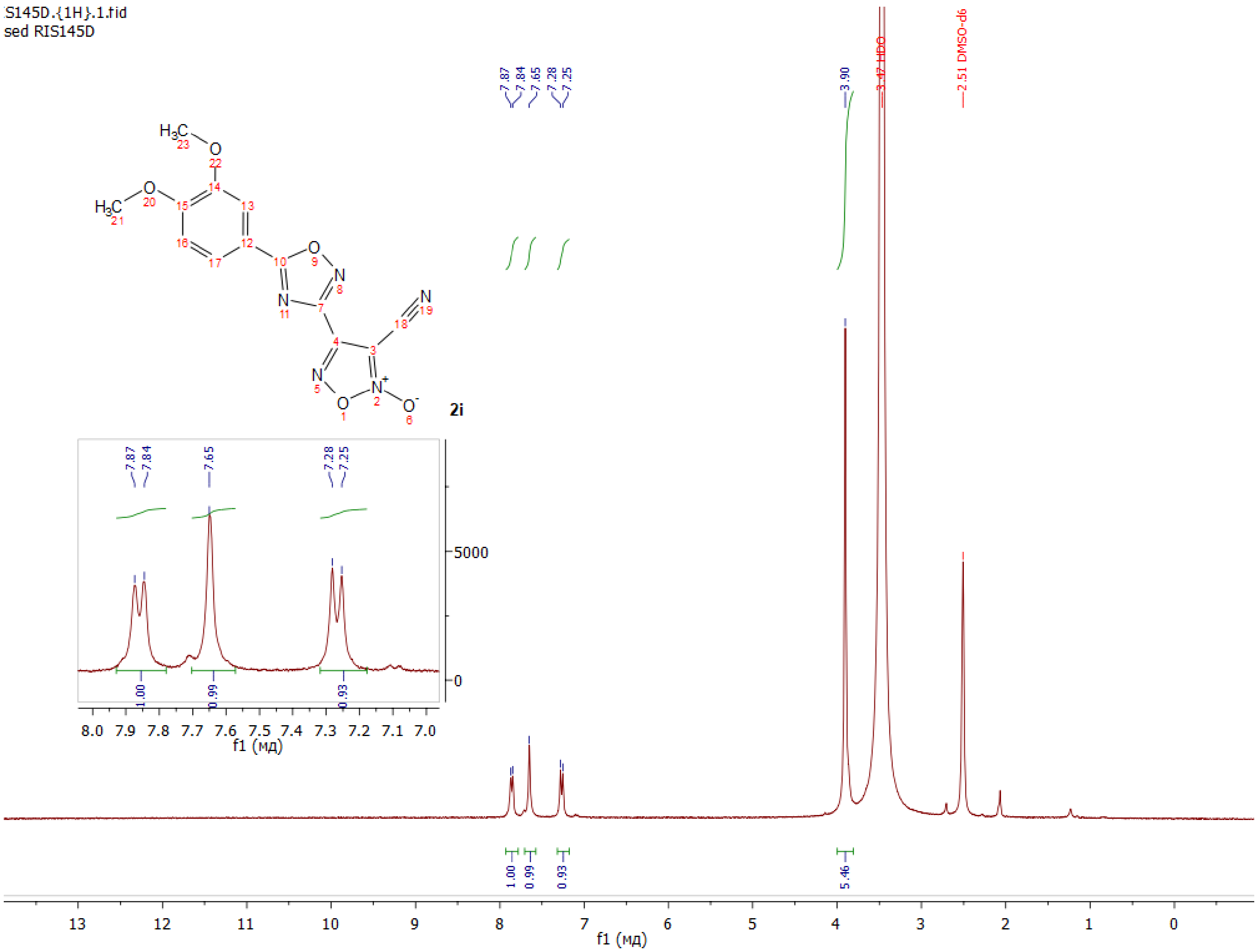

Figure S1.17. ^{1}H NMR spectrum of **2i**, DMSO-[d$_6$]

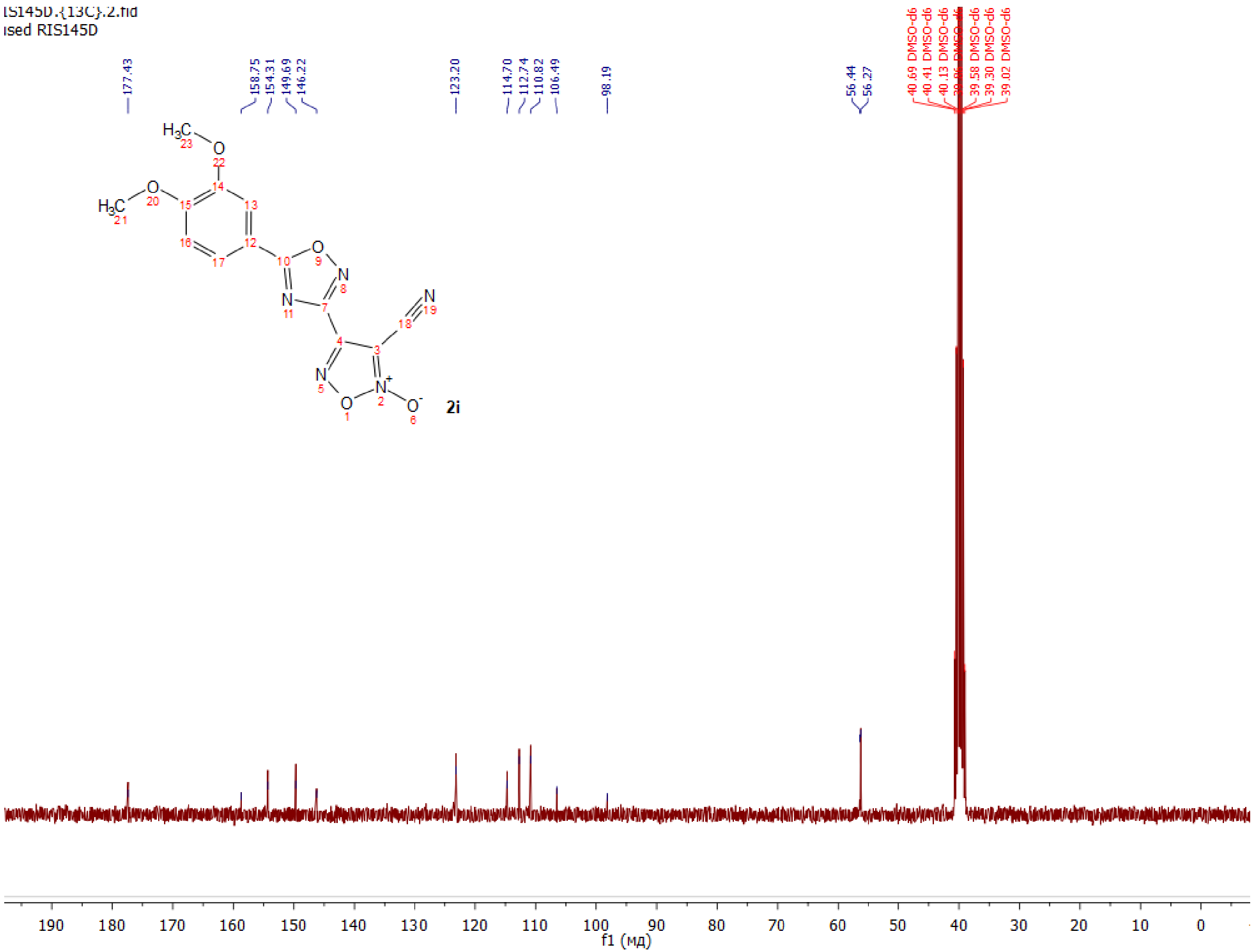

Figure S1.18. ^{13}C NMR spectrum of **2i**, DMSO-[d_6]

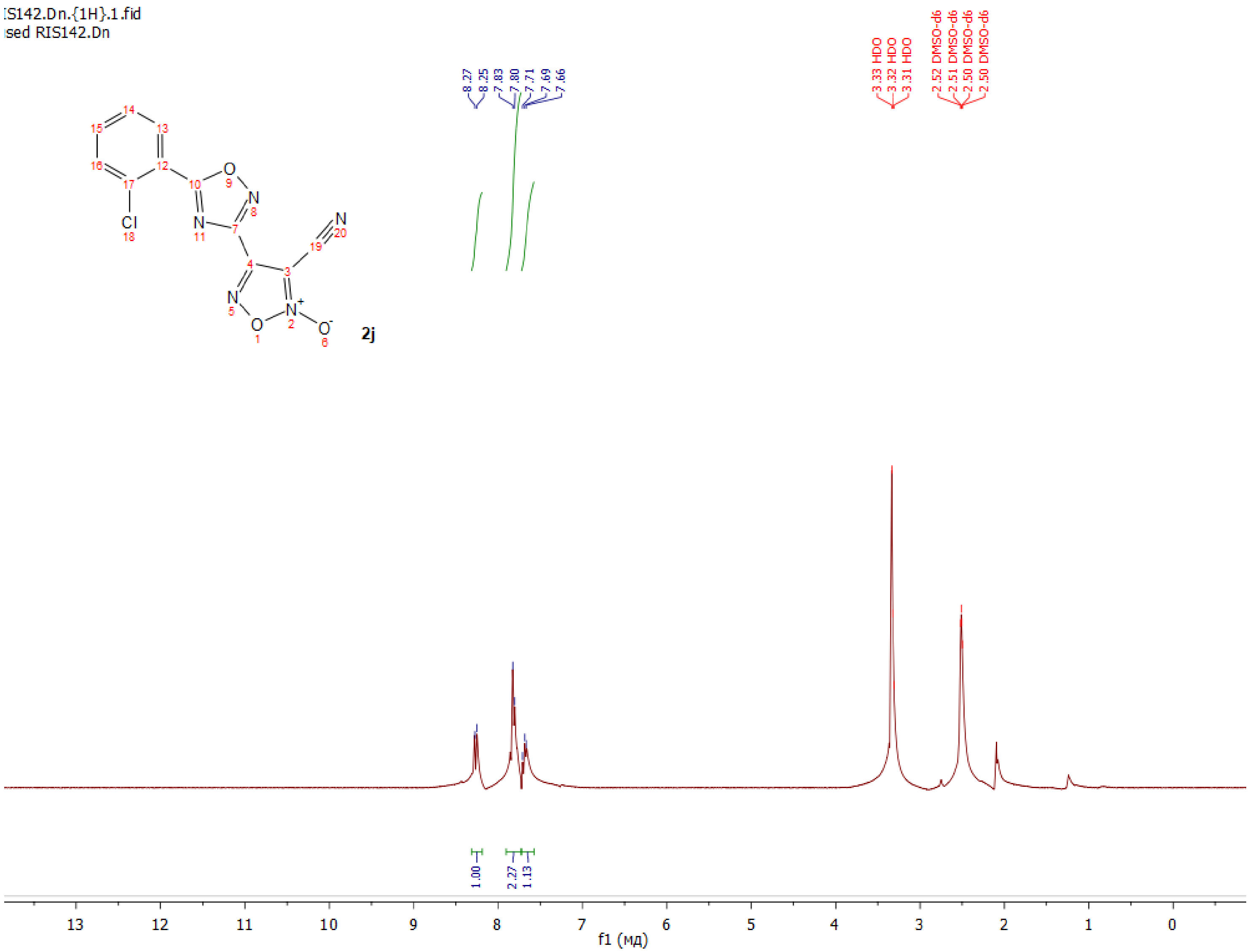

Figure S1.19. ^{1}H NMR spectrum of **2j**, DMSO-[d$_6$]

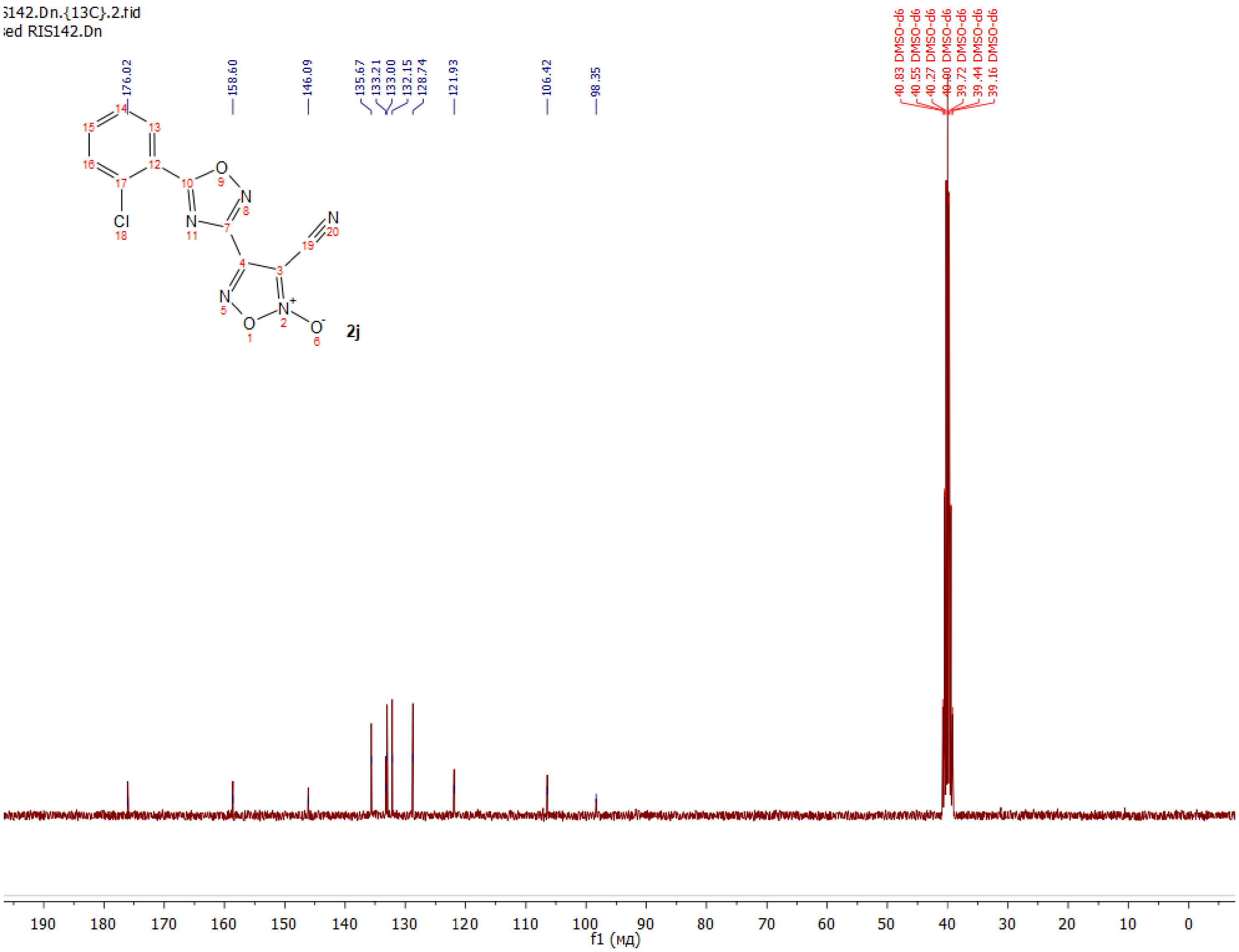

Figure S1.20. ^{13}C NMR spectrum of **2j**, DMSO-[d$_6$]

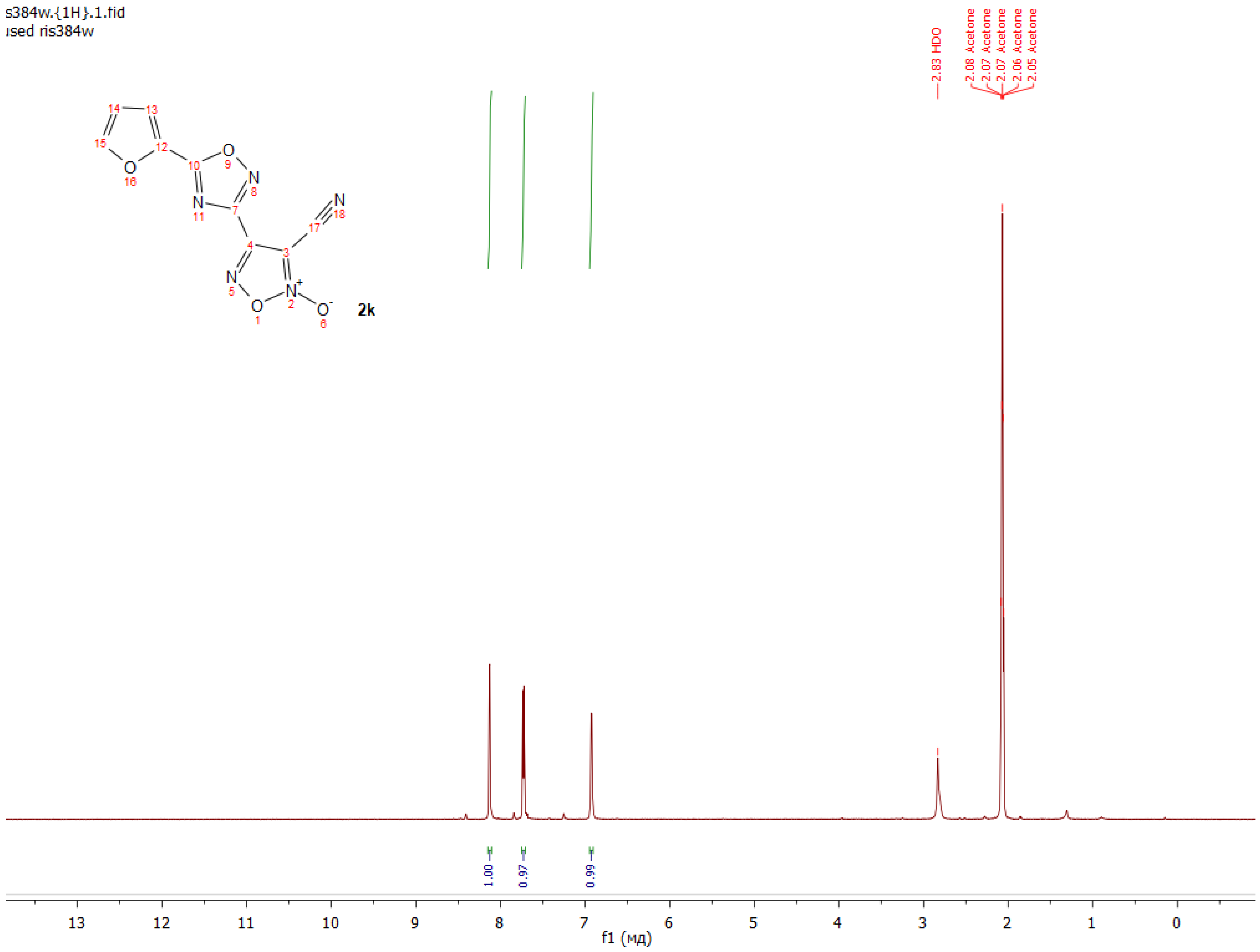

Figure S1.21. ^{1}H NMR spectrum of **2k**, Acetone-[d$_6$]

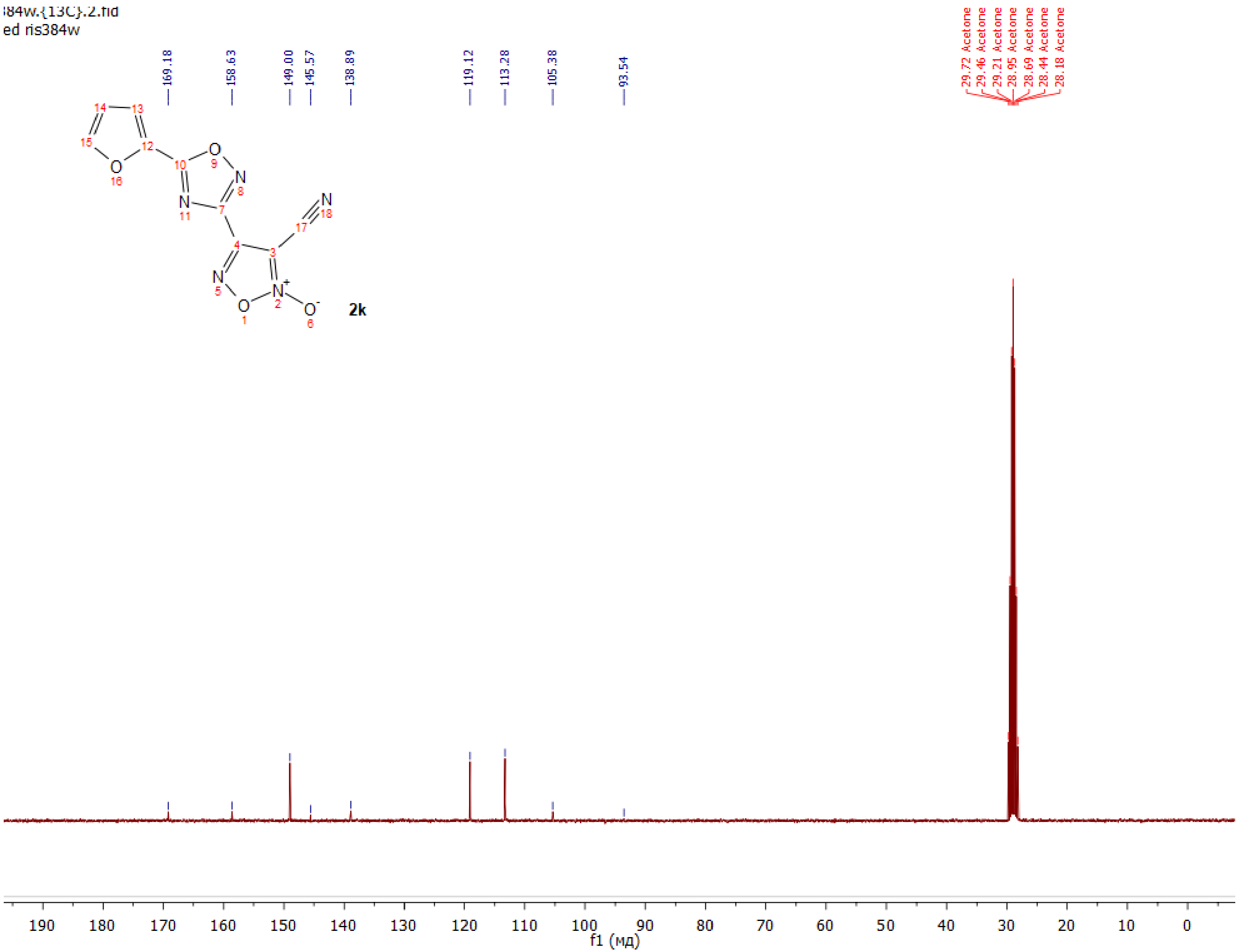

Figure S1.22. ^{13}C NMR spectrum of **2k**, Acetone-[d$_6$]

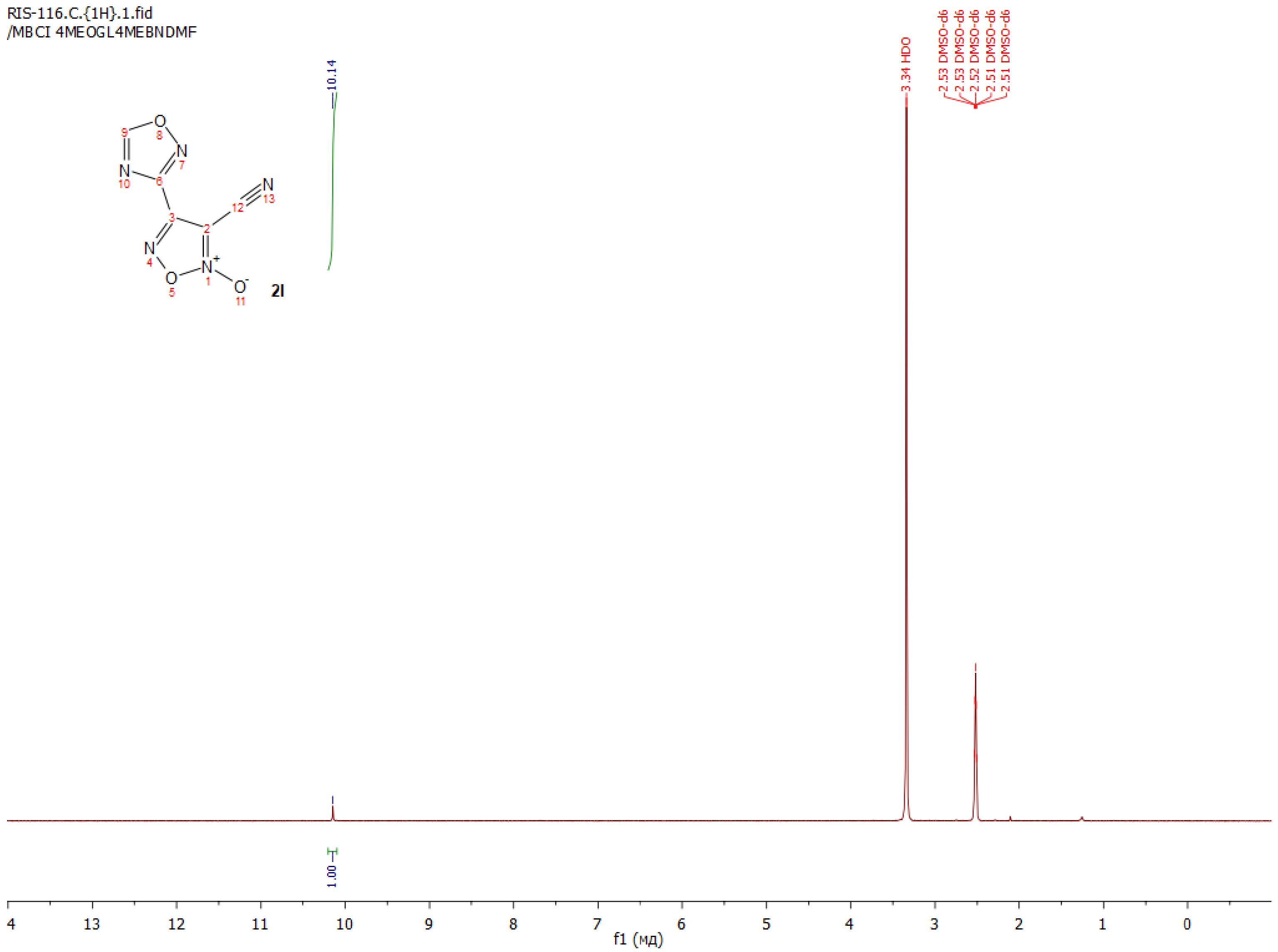

Figure S1.23. ^{1}H NMR spectrum of **2l**, DMSO-[d$_6$]

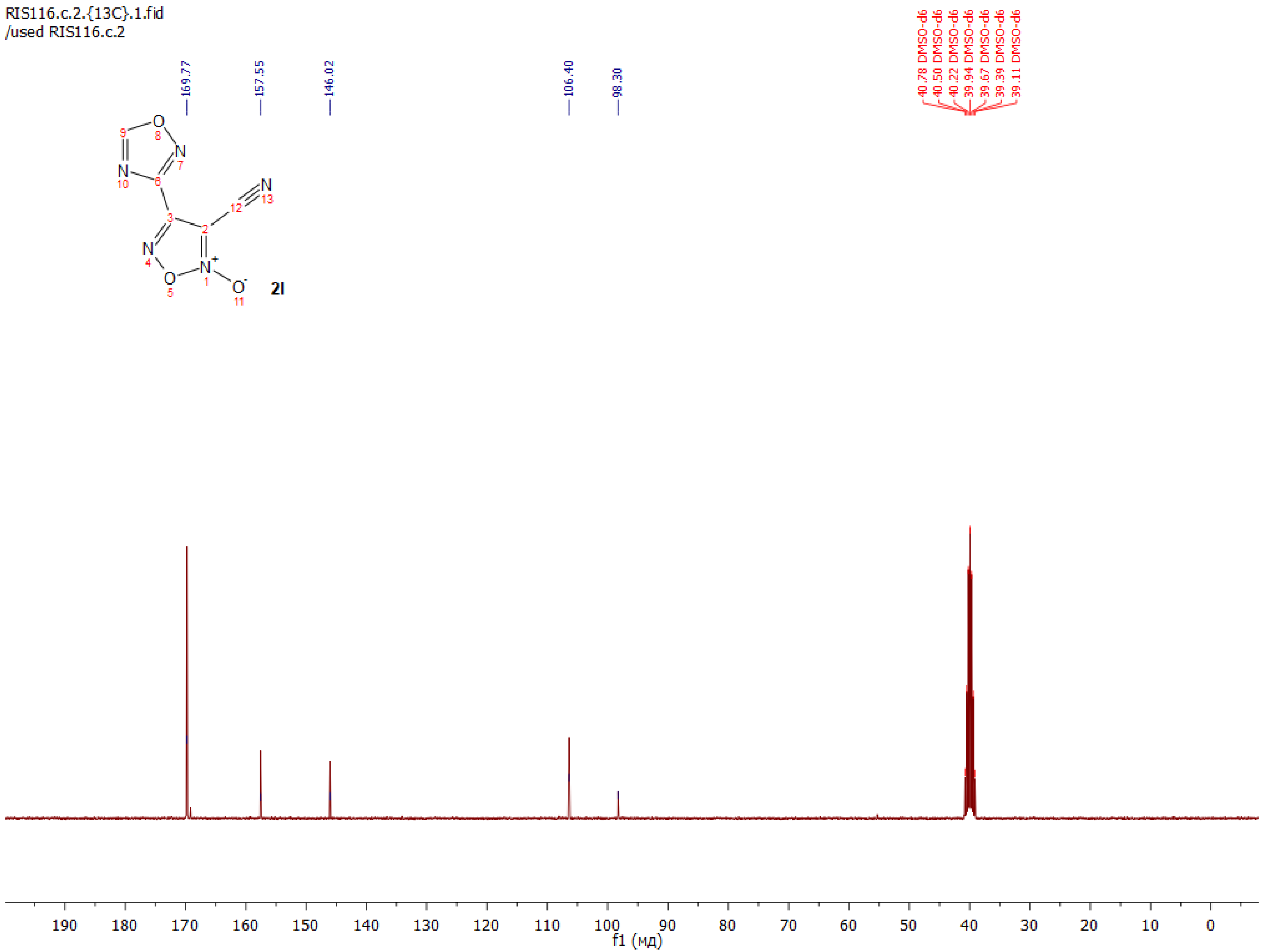

Figure S1.24. ^{13}C NMR spectrum of **2l**, DMSO-[d$_6$]

S2. Crystallographic data

Table S2.1. Main crystallography data and refinement details for the **2a** structure.

	**2a**
Formula	$C_{11}H_5N_5O_3$
Molecular mass	255.20
T, K	100
Crystal system	Orthorhombic
Space group	Pbca
Z (Z’)	8 (1)
a, Å	10.7518(2)
b, Å	8.16880(10)
c, Å	25.2967(4)
V, $Å^3$	2221.79(6)
d_{calc}, g·cm^{-3}	1.526
μ, cm^{-1}	1.17
F(000)	1040
$2\theta_{max}$, °	50
Number of reflections measured	30106
Independent reflections	3239
Reflections with I>2σ(I)	2862
Number of parameters	192
R1	0.0371
wR2	0.1014
GOF	1.040
Residual electron density, e·$Å^{-3}$(d_{min}/d_{max})	0.354/−0.287

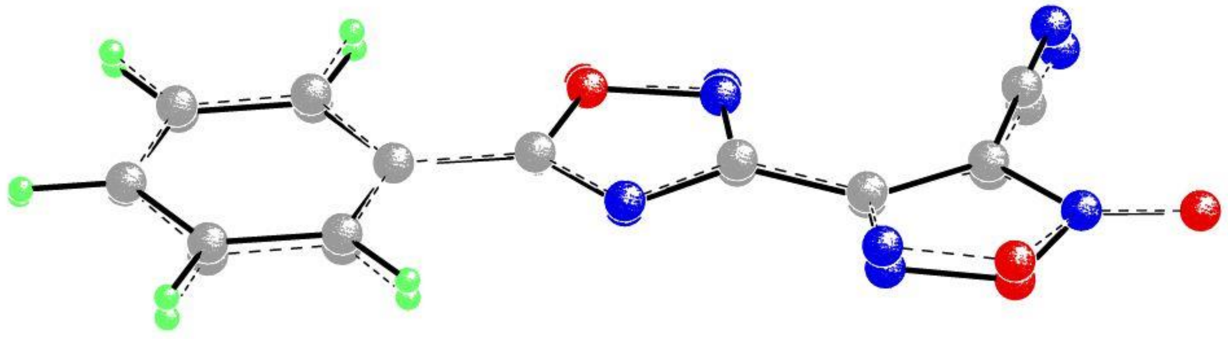

Figure S2.1. The best root mean square overlap of crystal (solid lines) and gas (dashed lines) conformations of **2a**.

S3. Biological activity

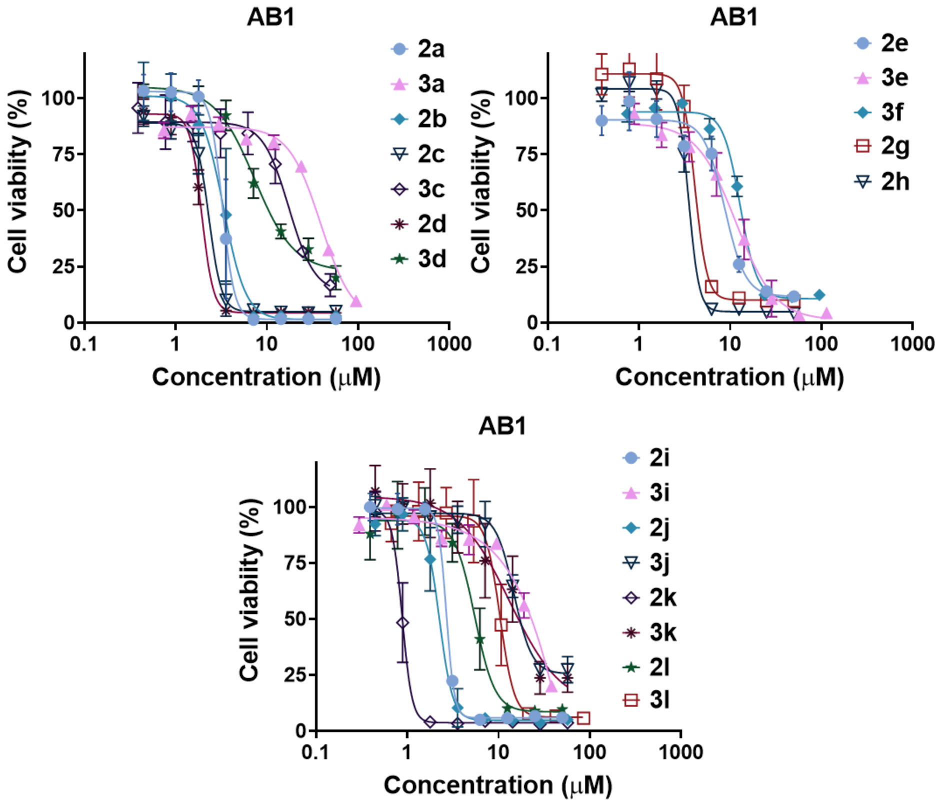

Figure S3.1 Concentration-effect curves for **2a-l** and **3a-l** in AB1 cell line upon 72 h exposure.

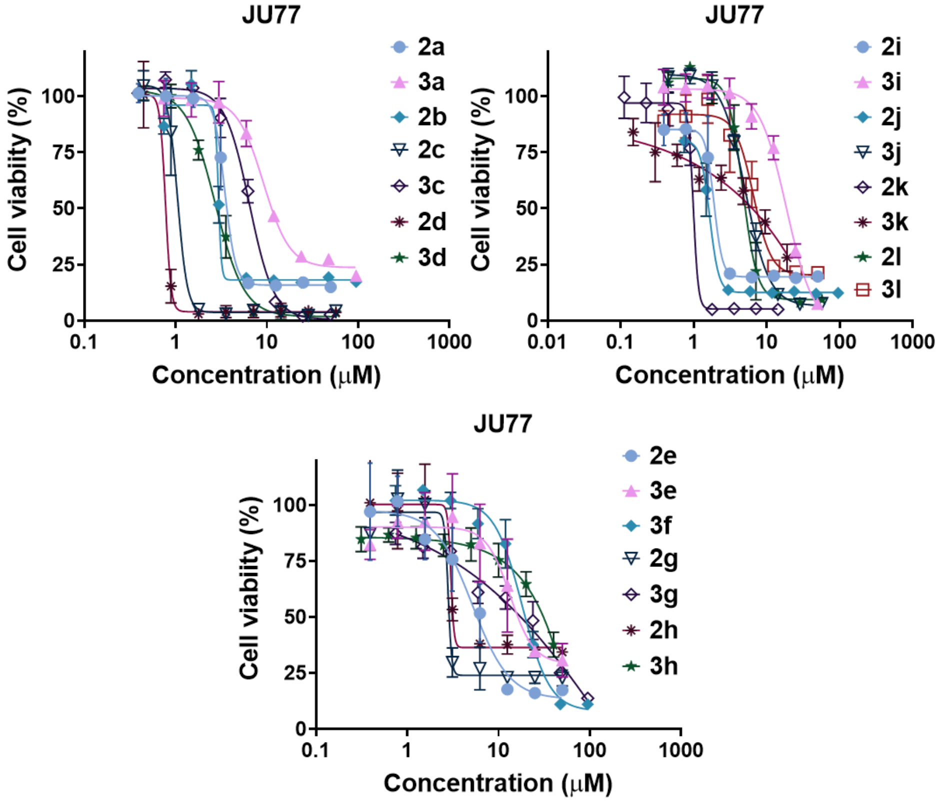

Figure S3.2. Concentration-effect curves for **2a-l** and **3a-l** in JU77 cell line upon 72 h exposure.

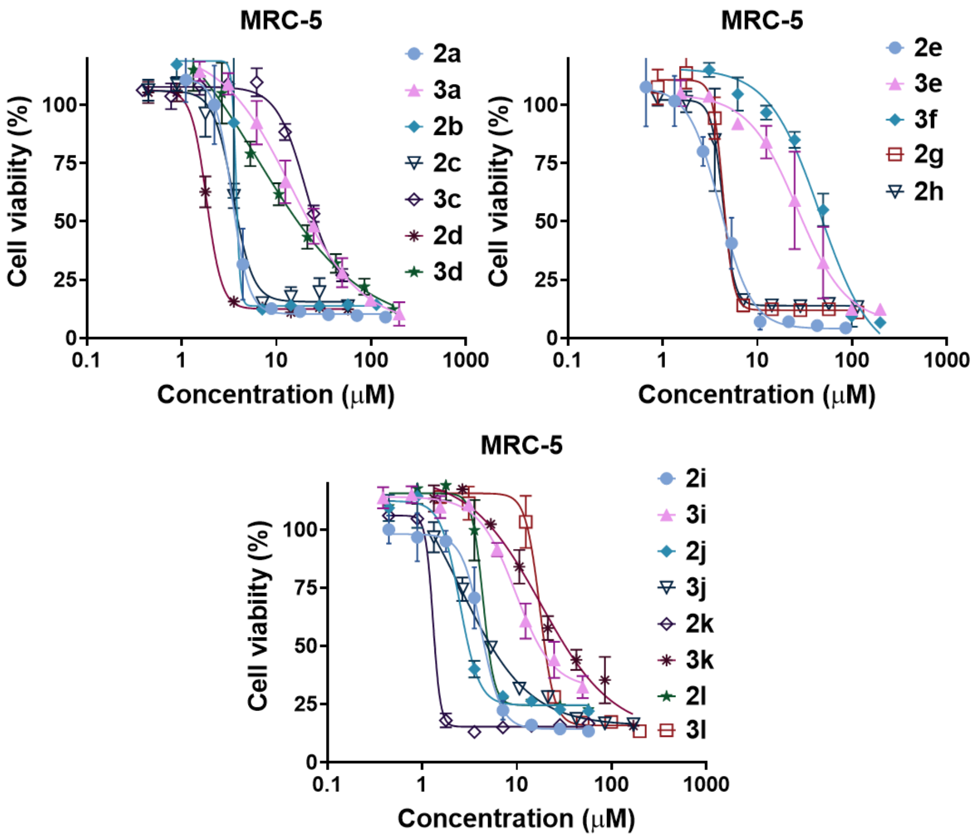

Figure S3.3. Concentration-effect curves for **2a-l** and **3a-l** in MRC-5 cell line upon 72 h exposure.
